# Identification of Novel Therapeutic Candidates Against SARS-CoV-2 Infections: An Application of RNA Sequencing Toward mRNA Based Nanotherapeutics

**DOI:** 10.3389/fmicb.2022.901848

**Published:** 2022-08-02

**Authors:** Zunera Khalid, Ma Huan, Muhammad Sohail Raza, Misbah Abbas, Zara Naz, Arnaud John Kombe Kombe, Weihong Zeng, Hongliang He, Tengchuan Jin

**Affiliations:** ^1^Department of Obstetrics and Gynecology, The First Affiliated Hospital of University of Science and Technology of China (USTC), Division of Life Sciences and Medicine, University of Science and Technology of China, Hefei, China; ^2^CAS Key Laboratory of Genomic and Precision Medicine, Beijing Institute of Genomics, Chinese Academy of Sciences, Beijing, China; ^3^China National Center for Bioinformation, Beijing, China; ^4^University of Chinese Academy of Sciences, Beijing, China; ^5^CAS Key Laboratory of Innate Immunity and Chronic Disease, School of Basic Medical Sciences, Division of Life Sciences and Medicine, University of Science and Technology of China, Hefei, China; ^6^Department of Infectious Diseases, The First Affiliated Hospital of University of Science and Technology of China (USTC), Division of Life Sciences and Medicine, University of Science and Technology of China, Hefei, China; ^7^CAS Center for Excellence in Molecular Cell Science, Shanghai, China

**Keywords:** SARS-CoV-2, PBMCs, RNA sequencing, upregulated genes, mRNA based nanotherapeutics, COVID-19 management

## Abstract

Due to fast transmission and various circulating SARS-CoV-2 variants, a significant increase of coronavirus 2019 infection cases with acute respiratory symptoms has prompted worries about the efficiency of current vaccines. The possible evasion from vaccine immunity urged scientists to identify novel therapeutic targets for developing improved vaccines to manage worldwide COVID-19 infections. Our study sequenced pooled peripheral blood mononuclear cells transcriptomes of SARS-CoV-2 patients with moderate and critical clinical outcomes to identify novel potential host receptors and biomarkers that can assist in developing new translational nanomedicines and vaccine therapies. The dysregulated signatures were associated with humoral immune responses in moderate and critical patients, including B-cell activation, cell cycle perturbations, plasmablast antibody processing, adaptive immune responses, cytokinesis, and interleukin signaling pathway. The comparative and longitudinal analysis of moderate and critically infected groups elucidated diversity in regulatory pathways and biological processes. Several immunoglobin genes *(IGLV9-49, IGHV7-4, IGHV3-64, IGHV1-24, IGKV1D-12*, and *IGKV2-29)*, ribosomal proteins *(RPL29, RPL4P2, RPL5*, and *RPL14)*, inflammatory response related cytokines including Tumor Necrosis Factor *(TNF, TNFRSF17*, and *TNFRSF13B)*, C-C motif chemokine ligands *(CCL3, CCL25, CCL4L2, CCL22*, and *CCL4)*, C-X-C motif chemokine ligands *(CXCL2, CXCL10*, and *CXCL11)* and genes related to cell cycle process and DNA proliferation (*MYBL2, CDC20, KIFC1*, and *UHCL1*) were significantly upregulated among SARS-CoV-2 infected patients. 60S Ribosomal protein L29 *(RPL29)* was a highly expressed gene among all COVID-19 infected groups. Our study suggested that identifying differentially expressed genes (DEGs) based on disease severity and onset can be a powerful approach for identifying potential therapeutic targets to develop effective drug delivery systems against SARS-CoV-2 infections. As a result, potential therapeutic targets, such as the RPL29 protein, can be tested *in vivo* and *in vitro* to develop future mRNA-based translational nanomedicines and therapies to combat SARS-CoV-2 infections.

## Graphical Abstract

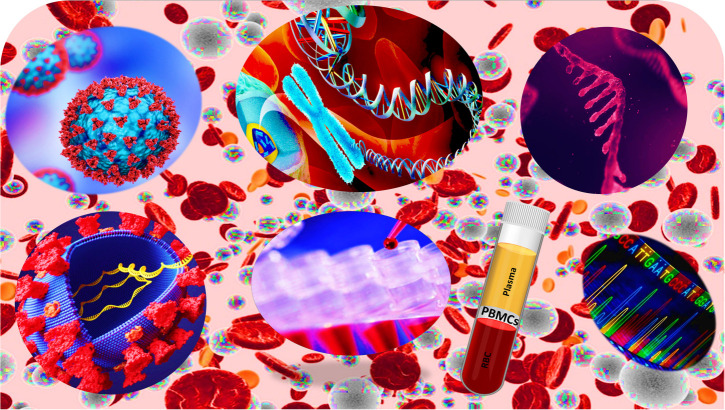

## Introduction

The main virus driving the COVID-19 (coronavirus disease 2019) pandemic, SARS-CoV-2 (Severe Acute Respiratory Syndrome Coronavirus 2), is highly contagious and can cause a wide spectrum of infections. This death-causing pneumonia epidemic has resulted in 452,201,564 cases and 6,029,852 fatalities worldwide, according to WHO^1^ (Liu T. et al., 2020). The symptoms, including dry cough, fever, sneezing, and malaise, were detected in patients with mild and moderate infections (Chen G. et al., 2020; Huang et al., 2020; Wang D. et al., 2020). In contrast, the severe patients are marked with ALI (Acute Lung Injury), ARDS (Acute Respiratory Distress Syndrome), pneumonia, and multiple organ failure (Liu T. et al., 2020). The older age groups also possess comorbidities, including hypertension, diabetes, cardiac diseases, hypoxia, and angiogenesis (Fung and Babik, 2020; Zhou et al., 2020). According to SARS-CoV-2 hospitalization data, the acute illness phase disproportionately infects elderly individuals and those with pre-existing comorbidities (Ahmed-Hassan et al., 2020; Cummings et al., 2020).

The literature shows that a dysregulated host inflammatory response plays a significant role in the morbidity and death of the viral disease (Lucas et al., 2020). When the virus infects a cell, it has the potential to activate and degrade the adaptive and innate host immune responses, which are critical components of the defense against viral invasion (Cao et al., 2021). SARS-CoV-2 infections lead to massively complicated metabolic pathways and cellular processes, and it is becoming progressively clear that the infected organism’s immune system has a significant effect on the disease progression (Islam et al., 2021). Moreover, immune profiling of moderate and critical patients using mass spectrometry and transcriptome/RNA sequencing has revealed monocyte-derived inflammatory macrophages, cell cycle perturbations, impaired G1/S phase transitions, DNA proliferation, T-cell responses, reduction in natural killer cells (NK), high neutrophil to lymphocyte (NLR) ratio, elevated expression of growth factors, and a delayed IFN response leading to the immune dysfunctioning (Blanco-Melo et al., 2020; Kuri-Cervantes et al., 2020; Zhang et al., 2020; Zheng H. Y. et al., 2020; Zheng M. et al., 2020). The signaling pathways driven by *IL-6*, *IL-1*β, and *TNF-*α have remained implicated in SARS-CoV-2 pathogenesis (Vabret et al., 2020), and antibodies against the *IL-6* receptor have shown early promise. The severity and magnitude of such inflammatory responses have emphasized scientists’ interventions that modulate the immune responses in SARS-CoV-2 infected patients from corticosteroids to specific cytokine inhibitors (Guo et al., 2020; Price et al., 2020).

In contrast to early findings that identified a cytokine storm-associated inflammatory response as a distinct feature of SARS-CoV-2-induced infections, current research using extensive profiling of host immune responses and more comprehensive cohort data suggests that a hyper-activated inflammatory response and abnormally repressed antibody-mediated immune signatures are a leading cause of fatal infections in COVID-19 patients (Lombardi et al., 2020; Unterman et al., 2022). Moreover, SARS-CoV-2 belongs to an RNA family. Therefore, the recent findings suggest that the first encoded viral protein virulence factor of SARS-CoV-2 named *NSP1* (non-structural protein 1) inhibits human mRNA translation by binding with human ribosomal proteins, disrupting the protein’s translational mechanism (Lapointe et al., 2021). Such inconsistent results might originate due to variations in disease severity, the onset of infection diagnosis, environmental conditions, and several other consequences that may differ across studies. Considering that SARS-CoV-2 is a fatal pathogenic disease, it is still significant to investigate alterations in the host immune systems based on disease severity and onset to identify targeted host receptors that can be helpful in designing more effective and standard drug therapies for COVID-19 patients.

Recent advances in sequencing technologies and multi-omics investigations have made it easier to employ genomes, transcriptomics, and proteomics data to investigate complicated biological processes and host-pathogen interactions. The yield from NGS (Next-generation sequencing) platforms has exceeded the order of terabytes (and billions of sequencing reads). Therefore, it has become easier and essential to interpret and visualize the data related to nano-biomaterials using best practices such as RNA sequencing, metabolic pathway associated transcriptome studies, and single-cell RNA studies (Paunovska et al., 2019). Nanotechnology tools play an essential role in advancing SARS-CoV-2 treatments and vaccine production. Current therapeutics focus on the complex molecular interactions in viral infections devoid of certain antivirals against SARS-CoV-2 (Varahachalam et al., 2021). Nanomaterials provide an emergent platform to improve diagnostic carriers for a vaccine and therapeutic development because of their distinctive size, tunable charge, low toxicity, and chemical modification abilities (Gage et al., 2021). They can bind with target host receptors and bioactives to establish a measurable signal that allows identification and detection of the virus (Bidram et al., 2021; Chaudhary et al., 2021; Gage et al., 2021). The distribution spectrum of nanocarriers becomes critical because most COVID19 vaccine candidates are intricate biological moieties (DNA, mRNA, engineered APCs, recombinant proteins) (Varahachalam et al., 2021). Identifying mRNA-based therapeutic targets employing omics studies is easy to operate and has accurate, stable, and susceptible landscapes, which can help design more effective nanotechnology-based mRNA therapeutics for several viral infections, including SARS-CoV-2. Therefore, nanotechnologists and omics-data analysts should work together to understand molecular biology, data analysis, and data visualization by innovative technologies to combat deadly pathogens and SARS-CoV-2.

The most effective COVID-19 vaccines are composed of mRNA derived from SARS-CoV-2 cell surface proteins and are encapsulated into nanoliposomes with specific physicochemical characteristics (Rashidzadeh et al., 2021). The studies have shown that mRNA-based vaccinations produce better humoral host responses. The splendid improvement toward the SARS-CoV-2 research and diagnostic treatments is the development of fast-tracking approved nanotechnology-based SARS-CoV-2 mRNA vaccines from Moderna and Pfizer/BioNTech employing lipid nanoparticles (Chaudhary et al., 2021; Milane and Amiji, 2021) and 77 additional vaccines in fast-tracked trials are also nanotherapeutics (MCVTJAohctov Team, 2021). In principle, an mRNA vaccine is composed of synthetic mRNA molecules encoded with an immunogenic sequence that instructs the cell’s ribosomal machinery to synthesize vaccine protein antigens and triggers the host immune response. Once the vaccine is transported to the cells, the ribosomes decode the mRNA vaccine sequence and generate the antigenic SARS-CoV-2 spike protein. The spike protein subsequently initiates the immunological response, including antibody synthesis and cellular immune responses (Chaudhary et al., 2021).

Entirely 57.2% of the worldwide population has been fully vaccinated (see text footnote 1). Still, with the emergence of the new variants, SARS-CoV-2 has become a primary epidemiological, virological, and clinical concern, predominantly concerning the risk of escape from the vaccine-induced immunity (Hastie et al., 2021; Tao et al., 2021; Colson et al., 2022). The Omicron (B.1.1.529.1) variant, first reported on November 24, 2021, has rapidly been recognized as the fifth Voc (a variant of concern) and has potentially spread globally (Karim and Karim, 2021). The widespread infections associated with the Omicron variant circulating even among doubly vaccinated individuals have confounded virologists, infectious disease specialists, and epidemiologists as the mutated variants are expected to be more transmissible and infectious than existing deadly Delta variants of the virus and are potentially invasive toward the cutting-edge therapeutics approaches, including vaccines (Mostafavi et al., 2022). Concerns about vaccine efficacy being harmed by new variations have shifted our perspective on the COVID-19 endpoint, casting doubt on the assumption that global vaccination is adequate to prevent SARS-CoV-2 infections (Karim and Karim, 2021).

To tackle this devastating pandemic, scientists still struggle to comprehend the host immune responses and biological processes to uncover new possible treatments and vaccine targets. In the current study, we opted for the total RNA sequencing techniques to identify the key regulators responsible for the dysregulated adaptive immunity of SARS-CoV-2 patients using the peripheral blood mononuclear cells (PBMCs) transcriptome based on their disease severity and progression. We evaluated the differentially expressed genes (DEGs), host-pathogen interactions, and regulatory networks interrupted during COVID-19 progression. The transcriptome data obtained from the current study has contributed to identifying novel therapeutic targets to design mRNA-based translational nanomedicines and vaccine therapies against SARS-CoV-2.

Patients with COVID-19 symptoms and diagnosis exhibit dysregulated immune signatures accompanying the humoral immunity, including B-cell receptor signaling pathway, cell cycle perturbations, DNA proliferation, plasmablast antibody processing, adaptive immune responses cytokinesis, and interleukin signaling pathway. Our findings also revealed that 60S Ribosomal L29 *(RPL29*) is a highly expressed gene in all COVID-19 infected groups, regardless of illness severity stage, implying a novel host receptor. Future mRNA-based translational nanomedicines and pharmacological therapies against SARS-CoV-2 infections can be proved *in vivo*.

## Materials and Methods

**FIGURE 1:**
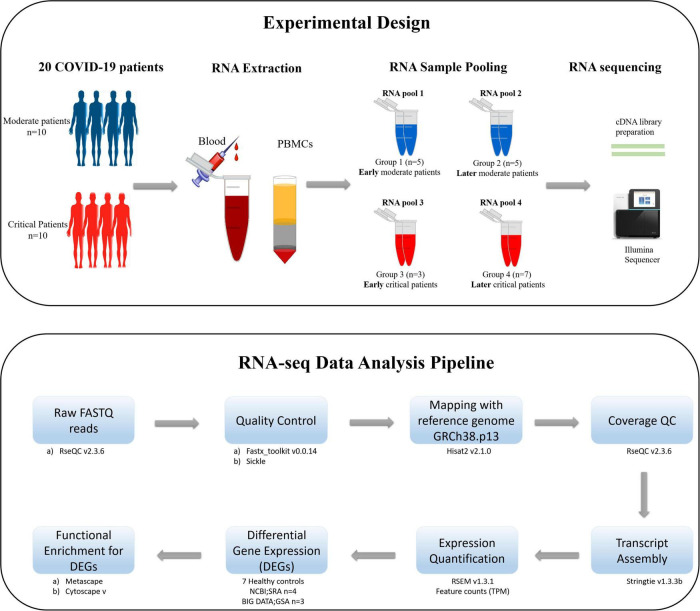
The schematic illustration of the pipeline adopted for the current study. The upper section of the figure illustrates the experimental design, isolation of PBMCs from the blood of COVID-19 patients, distribution of patients in each RNA pool, and sequencing method carried out for the current study. The lower section of the figure describes the data analysis pipeline, tools, and packages executed for this study.

The schematic diagram deciphering the experimental settings, grouping COVID-19 patients’ PBMC (Peripheral Blood Mononuclear Cell) samples into RNA pools, RNA sequencing (RNA-seq) of pooled samples, and sequencing data analysis pipeline adopted for the current study is illustrated in Figure 1.

### Settings and Ethics Statement

The current study was implemented on PBMC samples (Ficoll preparation) isolated from the whole blood of twenty COVID-19 patients obtained with informed consent on a protocol approved by the Medical Ethical Committee of The First Affiliated Hospital of the University of Science and Technology of China (USTC) (approval number 2020-XG (H)-019).

### Patients and Samples

The histopathology and baseline characteristics of the group of patients used in this study have been given in Supplementary File 1. According to the New Coronavirus Pneumonia Diagnosis and Treatment Plan (Trial Sixth Edition): the 10 patients had moderate symptoms such as fever, respiratory tract inflammation, and pneumonia diagnosed by imaging. At the same time, ten patients were diagnosed with acute COVID-19 symptoms based on lesion progression upon lung imaging, shortness of breath, higher breathing rate, lower oxygen saturation, and lower arterial blood oxygen partial pressure (Supplementary File 1).

### Preparation of Peripheral Blood Mononuclear Cells

The PBMC samples (Ficoll preparation) were extracted from the fresh blood of the twenty SARS-CoV-2 patients. Peripheral blood samples (4 ml) from each patient were drawn into vacutainer tubes by using Dipotassium (K2) Ethylene Diamine Tetraacetic Acid (EDTA) as an anti-coagulant within the vacutainers. The Ficoll 1.077 (Sigma Aldrich) density gradient centrifugation method separated the PBMC samples. The blood was diluted with 1 × phosphate-buffered saline (PBS) 1:1 and was shifted to the Ficoll tube. After centrifugation (20 min, 1,000 × g at room temperature), the buffy coat of PBMCs was pooled and moved into a 15-ml falcon tube. The cells were then washed twice with 10 ml PBS and centrifuged at 250 × g for 10 min.

### RNA Isolation and Sample Pooling

According to the manufacturer protocol of E.Z.N.A.^®^ Total RNA Kit I,^2^ the total RNA was extracted from PBMC samples by spin columns. The DNA was removed by the on-membrane DNase I digestion. Following the cost-effective RNA sample pooling strategy (Takele Assefa et al., 2020; Sawicki et al., 2021), the extracted RNA samples of all patients were pooled into four groups [Group 1: early moderate (*n* = 5), Group 2: later moderate (*n* = 5), Group 3: early critical (*n* = 7), Group 4: later critical (*n* = 3)] as illustrated in Figure 1. Patient groups’ early and later stages refer to the disease onset and diagnosis days when the SARS-CoV-2 virus infected the individuals for the first time. The early stage of patients groups refers to the first 15 days (average) after the disease onset, during which patients were at the acute phase of the infection, while the later stage of patients groups refers to the days from the 20th day after the disease onset during which patients were at the acute phase of the disease (Supplementary File 1).

The RNA concentration per sample sequencing was 50 ng/μl for Group 1 and Group 2, 500 ng/μL for Group 3, and 60 ng/μl for Group 4, respectively. The ratio of absorbance at 260 nm and 280 nm for all RNA samples was approximately 2.0. The cDNA libraries were prepared for the existing RNA samples, and the Illumina Novaseq 6000 platform performed total RNA sequencing for each RNA pool.

### RNA-Seq Data Analysis

The paired-end RNA-sequencing raw data (fastq format) obtained from Illumina sequencing were analyzed using the Differential Gene Expression pipeline (Figure 1). The RNA-seq data acquisition and interpretation comprise several steps; obtaining raw reads, read alignment and mapping, and expression quantification. Thorough checks should monitor the sequencing data’s quality and consistency at each stage. The actual analysis of RNA-seq data exhibits several discrepancies as the technology has several dimensions and applications. The primary research opted for the current RNA-seq experimental data involves quality control, read mapping and alignment with the reference genome, quantifying gene and transcript expression levels, and detecting DEGs among infected PBMCs RNA samples extracted from COVID-19 patients’ blood in comparison with the healthy PBMCs control RNA datasets. The downstream analysis involves global, comparative, and longitudinal GSEA (Gene Set Enrichment Analysis) of the selected DEGs from each infected group of patients.

#### Control Data Accessions

The seven RNA-seq datasets for PBMCs healthy control samples were recruited from the previous studies. The raw RNA sequencing data for four PBMCs healthy human donors (accession numbers: SRR1373441, SRR1373442, SRR1373453, and SRR1373454) were obtained from the NCBI SRA (Sequence Read Archive) database^3^ (Dvinge et al., 2014). The three PBMCs healthy donors (accession numbers: CRR125446, CRR125445, and CRR119890) were obtained from the BIG DATA GSA (Genome Sequence Archive)^4^ (Xiong et al., 2020).

#### Quality Control Checkpoints

FASTX-Toolkit-v0.0.14^5^ and fastp-v0.19.5^6^ is an ultra-fast fastq pre-processor, and it was utilized to calculate the quality checkpoints of the raw sequenced reads. The read quality reduces toward the 3’ end of the reads, and if it becomes too short, nucleotide bases should be removed to expand the mapping quality of the reads (Conesa et al., 2016). For accuracy, original sequencing data were filtered by SeqPrep,^7^ and the Sickle tool^8^ was used to remove low-quality reads, eliminate poor-quality bases, and trim adaptor sequences.

#### Reference Genome-Based Mapping

Hisat2-v2.1.0 alignment program^9^ with the default parameters was employed to align the cleaned RNA reads with the human (GRCh38.p13) reference genome.^10^ The percentage of aligned reads is a comprehensive mapping quality indicator referring to the sequencing accuracy and the presence of contaminated RNA. The quality evaluation of the mapped transcripts, including read distribution on different genome regions and read distribution at chromosomes, was done by RSeQC-2.3.6 (Wang et al., 2012).

#### Reference-Based Transcript Assembly Construction

Short RNA reads rarely span several splice junctions; therefore, it is challenging to infer all full-length transcripts directly. The aligned reads for each RNA pool were spliced and merged into potential transcripts by the stringtie-v1.3.3 assembler.^11^ The gffcompare program^12^ was adopted to compare, merge, annotate, and the transcript assembly when compared with reference genome for identifying similar and novel genes and transcripts.

#### Expression Quantification of Transcripts

The most straightforward approach for expression quantification is aggregating raw counts of mapped reads. RSEM (RNA-Seq by Expectation Maximization)^13^ was employed to estimate the gene and transcript expression levels. RSEM constructs a maximum likelihood abundance estimation method that depends on the highest expectation algorithm, considering paired-end reads, read length, fragment length distribution, and quality value, among other factors, to determine which transcripts are divergent isoforms of the same gene (Li and Dewey, 2011). This approach may distribute reads over multiple transcripts and generate within-sample normalized values corrected for sequencing biases (Conesa et al., 2016). Moreover, the RSEM algorithm uses an expectation-maximization method that returns TPM (Transcripts per million) reads which first normalizes the gene length and sequencing depth. The normalization process of TPM values marks the total expression in different samples consistent with comparing the gene expression more intuitively based on read counts.

#### Sample Correlation Analysis

The intergroup variability between experimental and control conditions referring to biological or technical variability is higher than the intragroup variability. Pearson’s correlation analysis was executed to identify the directionality and strength of the relationship between all samples. VENN diagram analysis displayed the unique and shared genes among the samples. PCA (Principal component analysis was performed to calculate the gene expression differences different among experimental conditions (control and infected). NOISeq-v2.18.0^14^ and Limma-v3.38.3^15^ were used to obtain exploratory plots to assess and visualize the samples’ expression matrix and differential expression.

#### Differential Gene Expression Analysis

The differential gene expression analysis was performed to compare and evaluate the DEGs between healthy controls and infected groups based on standard processing and screening conditions. As the current study consists of biological replicates from the same species *(Homo sapiens)*, R package DESeq2-v1.24.0^16^ was utilized, which integrates normalized counts data (TPM) to expedite the quantitative analysis of RNA-seq data employing fold change (Fc). The default parameters for screening significant DEGs were p-adjust < 0.05 and | log2fc| ≥ 1.

#### Functional Enrichment and Interactome Analysis of Selected Differentially Expressed Genes *via* Metascape

Functional enrichment and pathway analysis of selected DEGs among the samples were done by Metascape^17^ multiple gene set analysis. Metascape is an efficient annotation and regulatory pathway predicting tool. It applies prevalent bioinformatics analysis techniques for analyzing batch genes and proteins to reflect their functions (Zhou et al., 2019) by integrating numerous authoritative, functional databases such as GO (Gene Ontology) (Dimmer et al., 2012) and KEGG (Kyoto Encyclopedia of Genes and Genomes) (Kanehisa and Goto, 2000) for analyzing the human as well as other species data. The obtained regulatory networks and interactomes were visualized and analyzed by Cytoscape-v2.8 (Smoot et al., 2011), an open visualization software tool. The Cytoscape plug-in known as MCODE (Molecular Complex Detection tool) (version 1.5.1) (Bader and Hogue, 2003) was utilized to screen and identify the most significant modules in the Interactome of proteins, with the threshold values of MCODE scores > 5, node score cut-off = 0.2, k-score = 2, the degree of cut-off = 2, and maximum depth = 100.

## Results

In the present study, we sought to gain deeper insights into the host immune responses toward SARS-CoV-2 infections across disease severity stages and onset. We adopted a transcriptomics-based approach to evaluate four pooled RNA groups (early moderate, later moderate, early critical, and later critical) of PBMC samples isolated from twenty COVID-19 patients (Figure 1).

The sequenced RNA-datasets of COVID-19 infected PBMC samples were allocated into two bands: “Group” and “Control” where “Group” refers to the four infected patients’ RNA pools (Group 1, Group 2, Group 3, Group 4) which are sequenced for the current study (Figure 1) and “Control” refers to the seven previously published healthy PBMCs RNA-seq datasets (see section “Materials and Methods”).

As the study consists of pooled RNA samples of COVID-19 patients, a “control” cohort for entire healthy individual datasets was generated to obtain statistically significant outcomes of DEGs.

### Transcriptome Data Preprocessing and Quality Control

PBMC samples from all four COVID-19 infected groups and seven healthy controls were used to prepare RNA-seq datasets with 7.95E ± 07 million raw reads per specimen and 8.07E ± 09 million raw bp (base pairs). In contrast, an average of 7.78E ± 07 million clean reads and 7.77E ± 09 million clean bases were obtained after quality control checks and trimming of the adapter sequences (Supplementary Table 1). For each RNA-seq dataset, ∼90% of the total reads were uniquely mapped to the reference human genome GRCh38.p13 (see text footnote 10) (Supplementary Table 2).

Gene prediction should ideally identify all exons and introns, including those in the Mrna’s 5′-UTR and the 3′-UTR (Untranslated regions), to appropriately reconstruct the dominant mRNA species. However, it is beneficial to accurately assemble the coding exons (CDs) to determine the protein sequence for a practical purpose. The gene coverage ratio and homogeneity distribution analysis provided insights into the distribution of the expected reads within and across the genes. It was also suggested that maximum genes ∼ 63.81% of the reads were mapped to exonic regions of the reference sequence (Supplementary Figure 1). The gene coverage and chromosome distribution of all RNA-Seq datasets revealed a more significant similarity in mapping density across different chromosomes for each sample (Supplementary Figure 2).

### Expression Quantification of Peripheral Blood Cells Transcriptome

**FIGURE 2:**
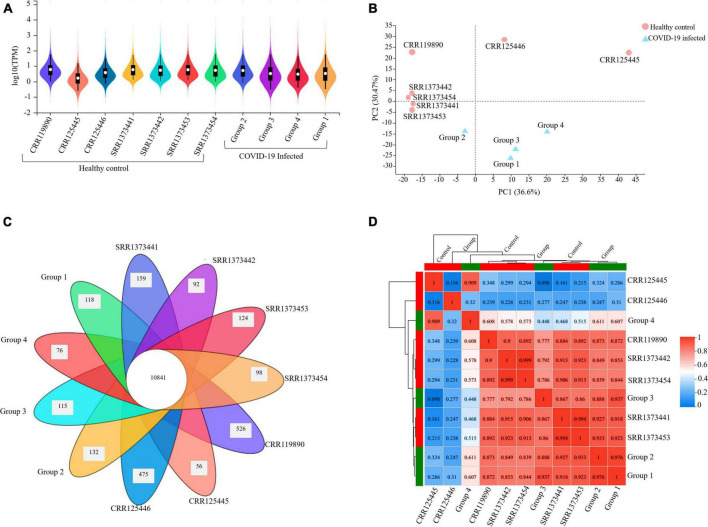
Gene expression quantification and sample correlation of PBMC samples. **(A)** The violin map illustration of expression distribution in each sample (X-axis) is based on the log10 (TPM) (Y-axis), which is the parametric value of the expression level. **(B)** PCA (Principal Component Analysis) plot presenting entire samples along PC1 (X-axis) and PC2 (Y-axis), describing 36.6 and 30.4% of the variability within the expression data set. PC analysis practiced normalized (reads per kilobases of transcript per 1 million mapped reads) and log-transformed count data. The closer the distance of each sample, the higher the similarity between the samples. **(C)** Venn diagram illustrating the number of co-expressed genes among all samples at the intersection (10841) and the number of uniquely expressed genes in each individual that are not part of this common set. **(D)** Pearson’s correlation matrix visualizes the correlation values between samples with numbered Scale bar representing the range of the correlation coefficients.

The expression profile of the resulting transcript assembly detected 39,307 expressed genes (38,390 known and 917 new genes). The expression distribution violin map for each sample is illustrated in Figure 2A. The precision and accuracy of the results were also confirmed by testing the correlation and variability between samples based on the experimental conditions (COVID-19 infected groups) compared to the healthy control conditions. PCA (Principal Component Analysis) showed that 36.6% of the variation could be described by PC1 and 30.47% by PC2, primarily separating the infected and healthy groups. It was reflected by PCA analysis that biological replicates have a similar condition resulting in the clustering of the infected groups together and the dispersion of the other seven healthy samples, proposing that COVID-19 infected groups have variability in terms of expression level (Figure 2B). The Venn diagram analysis determined the number of co-expressed genes in all samples, representing the core of 10,841 common genes entirely expressed and a minimum of 56 and a maximum of 526 genes in each sample that were not a part of this common set (Figure 2C). The inter-sample clustering done by the Pearson correlation coefficient matrix illustrated a high consistency and similarity among all RNA-seq samples (Figure 2D). Therefore, the sample correlation analysis elucidated a more significant variability among the two conditions (infected and healthy), representing technical or biological variability.

### Global Transcriptome and Functional Enrichment Analysis of Cohort Data

To identify global signature pathways and genes in patients with SARS-CoV-2 infections, we combined the transcriptome data of all infected groups (early moderate, later moderate, early critical, and later critical groups) and their healthy controls. We made a comprehensive cohort called “Group” to compare against healthy controls cohort “Control” to identify DEGs (see section “Materials and Methods”). We identified 5,710 significant DEGs, including 1,101 significantly upregulated genes and 4,609 significantly downregulated genes (Supplementary File 1). Several immunoglobulin genes *(IGLV9-49, IGHV7-4, IGHV3-64, IGHV1-24, IGKV1D-12*, and *IGKV2-29)*, ribosomal proteins *(RPL29, RPL4P2, RPL5*, and *RPL14)*, inflammatory response related cytokines including Tumor Necrosis Factor *(TNF, TNFRSF17*, and *TNFRSF13B*), C-C motif chemokine ligands *(CCL3, CCL25, CCL4L2, CCL22*, and *CCL4)*, C-X-C motif chemokine ligands *(CXCL2, CXCL10*, and *CXCL11)* were significantly upregulated among SARS-CoV-2 infected cohort in comparison with the healthy control cohort (Supplementary Figure 3 and Supplementary File 2).

**FIGURE 3:**
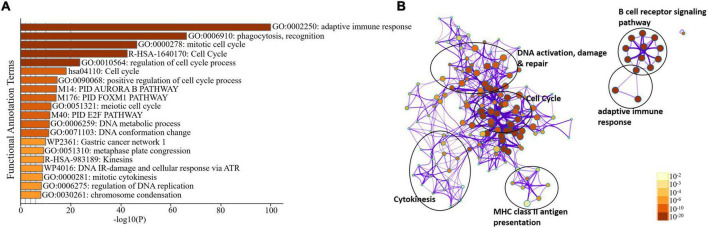
Enrichment analysis of differentially expressed genes in COVID-19 cohort. **(A)** Bar Graph representation of top twenty clusters with corresponding enriched terms across a provided list of DEGs colored by –log10(P) on the X-axis. The functional annotation terms and their corresponding categories are M (canonical pathways), GO (Gene Ontology), WP (Wiki pathways), R-HSA (Reactome Gene Sets), and hsa (KEGG pathway). **(B)** Functional enrichment analysis of DEGs compared between infected and healthy control groups illustrating the dysregulated regulatory pathways in COVID-19 cohort data. The network cluster labels are added manually. The nodes are represented as pie charts and colored by *p*-value, where enriched networks having more genes tend to have a higher *p*-value.

We sorted 500 upregulated DEGs obtained from the “cohort” based on their highest log2fc values and performed Gene Set Enrichment analysis *via* Metascape to interrogate signaling pathways and host immune responses induced by SARS-CoV-2 (Table 1 and Supplementary File 2). The upregulated DEGs were mainly enriched in adaptive immune response-related pathways, immunoglobulin production, B-cell receptor signaling pathways, PID aurora pathway, SARS-CoV-2 infection pathway, and several other infectious pathways (Figures 3A,B).

**TABLE 1:** Top 20 upregulated differentially expressed genes and their GO terms and fold change (log2fc) values.

No	Sequence ID	Gene name	Description	log2fc (Group/Control)	Padjust	GO term
**1**	ENSG00000230202	RPL29	Ribosomal protein L29 (RPL29) pseudogene	9.001	4.01E-25	**GO:0006660** phosphatidylserine catabolic process;**GO:0052651** monoacylglycerol catabolic process;**GO:0046462** monoacylglycerol metabolic process
**2**	ENSG00000183260	ABHD16B	Abhydrolase domain containing 16B	8.23	2.12E-15	**GO:0006660** phosphatidylserine catabolic process;**GO:0052651** monoacylglycerol catabolic process;**GO:0046462** monoacylglycerol metabolic process
**3**	ENSG00000262526	AC120057.2	Novel protein	7.95	0.036478	None
**4**	ENSG00000223350	IGLV9-49	Immunoglobulin lambda variable 9-49	7.21	4.77E-14	**GO:0002377** immunoglobulin production;**GO:0002440** production of molecular mediator of immune response;**GO:0002250** adaptive immune response
**5**	ENSG00000282122	IGHV7-4-1	Immunoglobulin heavy variable 7-4-1	6.79	2.30E-05	**GO:0006910** phagocytosis, recognition;**GO:0006958** complement activation, classical pathway;**GO:0002455** humoral immune response mediated by circulating immunoglobulin
**6**	ENSG00000253691	IGKV2OR22-4	Immunoglobulin kappa variable 2/OR22-4 (pseudogene)	6.60	2.28E-08	No Hit
**7**	ENSG00000256663	AC112777.1	Ubiquitin-like with PHD and ring finger domains 1 (UHRF1) pseudogene	6.56	7.00E-07	None
**8**	ENSG00000087116	ADAMTS2	ADAM metallopeptidase with thrombospondin type 1 motif 2	6.48	0.002565	**GO:0030574** collagen catabolic process;**GO:0030199** collagen fibril organization;**GO:0032963** collagen metabolic process
**9**	ENSG00000230699	AL645608.2	Novel transcript	6.38	5.72E-09	None
**10**	ENSG00000223648	IGHV3-64	Immunoglobulin heavy variable 3-64	6.24	4.01E-14	**GO:0006910** phagocytosis, recognition;**GO:0006958** complement activation, classical pathway;**GO:0002455** humoral immune response mediated by circulating immunoglobulin
**11**	ENSG00000277125	AC211476.4	PMS2 postmeiotic segregation increased 2 (S. cerevisiae) (PMS2) pseudogene	6.21	0.000115	None
**12**	ENSG00000211950	IGHV1-24	Immunoglobulin heavy variable 1-24	6.20	1.48E-13	**GO:0006910** phagocytosis, recognition;**GO:0006958** complement activation, classical pathway;**GO:0002455** humoral immune response mediated by circulating immunoglobulin
**13**	ENSG00000257027	AC010186.3	Novel transcript	5.78	4.74E-05	None
**14**	ENSG00000278857	IGKV1D-12	Immunoglobulin kappa variable 1D-12	5.74	5.39E-05	**GO:0002377** immunoglobulin production;**GO:0002440** production of molecular mediator of immune response;**GO:0002250** adaptive immune response
**15**	ENSG00000253998	IGKV2-29	Immunoglobulin kappa variable 2-29 (gene/pseudogene)	5.70	1.49E-15	**GO:0002377** immunoglobulin production;**GO:0002440** production of molecular mediator of immune response;**GO:0002250** adaptive immune response
**16**	ENSG00000211655	IGLV1-36	Immunoglobulin lambda variable 1-36	5.69	8.01E-16	**GO:0002377** immunoglobulin production;**GO:0002440** production of molecular mediator of immune response;**GO:0002250** adaptive immune response
**17**	ENSG00000211658	IGLV3-27	Immunoglobulin lambda variable 3-27	5.65	6.55E-32	**GO:0002377** immunoglobulin production;**GO:0002440** production of molecular mediator of immune response;**GO:0002250** adaptive immune response
**18**	ENSG00000211663	IGLV3-19	Immunoglobulin lambda variable 3-19	5.61	2.68E-13	**GO:0002377** immunoglobulin production;**GO:0002440** production of molecular mediator of immune response;**GO:0002250** adaptive immune response
**19**	ENSG00000211642	IGLV10-54	Immunoglobulin lambda variable 10-54	5.59	7.30E-22	**GO:0002377** immunoglobulin production;**GO:0002440** production of molecular mediator of immune response;**GO:0002250** adaptive immune response
**20**	ENSG00000232216	IGHV3-43	Immunoglobulin heavy variable 3-43	5.56	8.04E-18	**GO:0006910** phagocytosis, recognition;**GO:0006958** complement activation, classical pathway;**GO:0002455** humoral immune response mediated by circulating immunoglobulin

### Altered Transcriptome Profiles Across COVID-19 Severities

**FIGURE 4:**
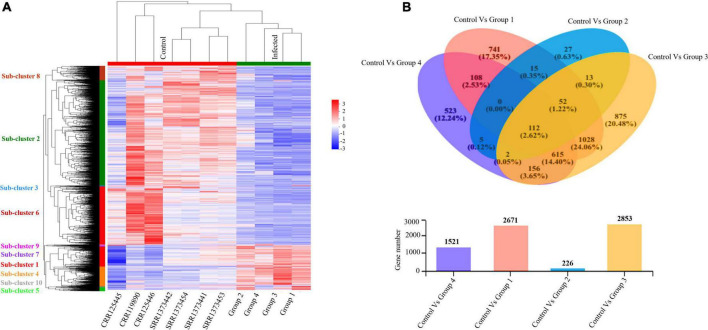
Altered transcriptome profiles across COVID-19 severities. **(A)** Hierarchal clustering Heat map for significantly upregulated and downregulated DEGs (fold change (log2FC ≥ 2) in COVID-19 PBMC samples compared to controls. The negative numbers having blue color indicate down-regulated genes, and the positive numbers with red color indicate upregulated genes. The sub-cluster labels on the Y-axis are added manually and specified by their corresponding color in the heat map. **(B)** Venn Diagram Analysis: Venn diagram of the healthy control cohort and each patient’s group. Circles with different colors represent the four infected Groups, and the number of genes/transcripts screened based on expression levels in each sample/group compared to the control cohort. The individual and overlapping portions in the Venn diagram illustrating the number of explicitly expressed and co-expressed genes among different groups.

Furthermore, we evaluated and compared DEGs to the healthy control cohort to determine whether the above global signatures and the identified upregulated genes and their functional enrichment pathways are similar or vary by disease severity stage and onset. Our results suggested a diverse transcriptome profile of DEGs in each group of patients based on the disease onset and severity. The DEGs are represented in the hierarchical clustering heat map depicting the significant differentially expressed gene clusters across each sample (Figure 4A). The expression pattern of genes was diversified across each sub-cluster corresponding to the clustering heat map (Supplementary Figure 4 and Supplementary Files 2, 3).

We detected 917 upregulated and 1,754 downregulated genes in group 1 (early moderate), 112 upregulated and 114 downregulated genes in group 2 (later moderate), 1,067 upregulated and 1,786 downregulated genes in group 3 (early critical), and 838 upregulated and 683 downregulated genes in group 4 (later critical) (Figure 4B and Supplementary File 4). When visualized as the scatter plots (Supplementary Figure 5), it was evident that a higher number of genes were upregulated in group 3 (early critical) while group 2 (later moderate) showed limited statistical significance.

### Functional Enrichment Analysis Across SARS-CoV-2 Severity Elucidating Diverse Host Immune Response Profile

**FIGURE 5:**
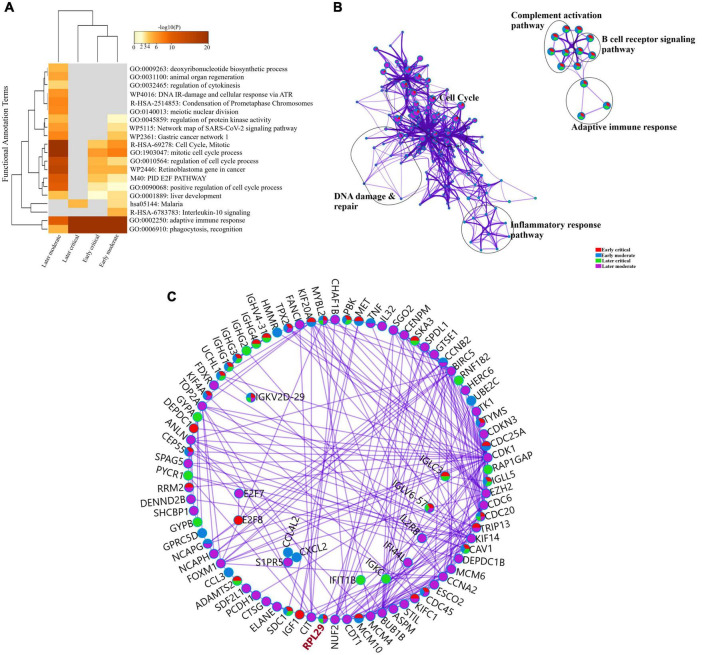
Functional enrichment analysis of multiple DEGs from all SARS-CoV-2 infected groups. **(A)** Heat map displaying the top 20 enriched clusters for early and later moderate and critical patients using a distinct color scale to represent statistical significance using -log10(P). The functional annotation terms and their categories are M (canonical pathways), GO (Gene Ontology), WP (Wiki pathways), R-HSA (Reactome Gene Sets), and hsa (KEGG pathway). Gray color designates the lack of significance. **(B)** Enrichment network visualization of multiple DEG lists. Cluster labels representing the network name were added manually. The clustered network showed that processes such as the B-cell signaling pathway, cytokinesis, interleukin signaling pathway, and cell cycle are shared between the early and later moderate gene lists. **(C)** Interactome analysis of proteins between DEGs of each group. Each color code describes the gene identities in the four COVID-19 groups.

To further delineate the differences among each SARS-CoV-2 infected RNA group, we sorted each group’s DEGs based on the highest log2fc value and lowest *p*-value. We picked the top 100 significantly upregulated DEGs from each infected RNA group and performed functional enrichment analysis. The maximum DEGs among all COVID-19 groups were highly enriched in adaptive immune response regardless of the disease onset and severity specific stage (Figure 5A and Supplementary File 4). The intergroup comparison reflected that DEGs in the early moderate, early critical, and later critical stages had dysregulated regulatory networks, including B-cell receptor signaling pathway, complement activation pathway, adaptive immune response, and phagocytosis compared to their healthy controls. Patients with early moderate symptoms were mainly enriched in cell cycle perturbations, DNA proliferation, and inflammatory response-related pathways (Figure 5B).

The Interactome analysis of several proteins based on regulatory networks elucidated a higher diversity of immune fluctuations across each severity stage. Upregulated gene *CCNB2* was identified in the moderate stage of patients, and it is the conventional marker of mitotic cells and regulates the cell cycle transition at the G2/M phase. Several upregulated genes (*KIF14, KIF4A, UCHL1*, and *KIFCI)* were involved in aberrant biological cell responses, including cell migration and adhesion, DNA damage, repair, and replication. Mesenchymal stem cells (MSCs) induced and upregulated *MYBL2* gene was present in all infected COVID-19 groups. Several upregulated immunoglobulin genes influenced the dysregulated antibody-mediated immune response. *IGKV2d-29* was upregulated in all groups, while *IGHG1* and *IGHG3* were upregulated in three infected groups except the later moderate stage. *CDC20* (cell division cycle 20) protein-coding gene was upregulated in all groups of patients (Figure 5C and Supplementary File 4). Therefore, comparative functional enrichment analysis of DEGs across each severity-specific stage suggested a diverse expression profile of each infected COVID-19 group.

The 60S Ribosomal Protein L29 *(RPL29)* was highly expressed among all SARS-CoV-2 infected RNA groups with an average fold change of 9 (Figure 5C and Supplementary File 4). The KEGG pathway analysis of *RPL29* protein revealed two pathways, “ribosome” and “Coronavirus pathway.” The detailed GO and KO annotation for the *RPL29* protein has been illustrated in Table 2.

**TABLE 2:** Functional Enrichment analysis of highly expressed RPL29 protein deciphering the GO terms and KEGG pathways.

Functional annotation	GO term	GO ID	GO description
GO	**Biological process**	GO:0000184	Nuclear-transcribed mRNA catabolic process, nonsense-
		GO:0002181	mediated decay
		GO:0006412	Cytoplasmic translation
		GO:0006413	Translation
		GO:0006614	Translational initiation
		GO:0007566	SRP-dependent cotranslational protein targeting to membrane embryo implantation
	**Cellular component**		
		GO:0019083	Viral transcription
		GO:0005829	Cytosol
		GO:0005840	Ribosome
		GO:0016020	Membrane
		GO:0022625	Cytosolic large ribosomal subunit
	**Molecular function**	GO:0003723	RNA binding
		GO:0003735	Structural constituent of ribosome
		GO:0008201	Heparin-binding
		GO:0045296	Cadherin binding
KO	**KO ID**	**KO name**	**KEGG pathway**
	K02905	RP-L29e, RPL29	Map03010: Ribosome map05171: Coronavirus disease—COVID-19

### Longitudinal Analysis of Early and Later Disease Onset of COVID-19 Severity

The longitudinal studies of RNA-seq data are essential for differentiating variations among different samples, and gene expression repeatability to decipher gene and transcript variants (Rondina et al., 2020). In the current study, we also performed longitudinal analysis to examine within (intra-) and between (inter-) sample variability of the PBMCs transcriptome of infected groups based on their early and later disease onset with respect to the healthy controls.

The early and later stages of patient groups refer to the days of the disease onset of the COVID-19 diagnosis when the SARS-CoV-2 virus infected the individuals for the first time. The early stage of patients groups refers to the first 15 days (average) after the disease onset, during which patients were at the acute phase of the infection, while the later stage of patients groups refers to the days from the 20th day after the disease onset during which patients were at the acute phase of the disease (Supplementary File 1). We explored diverse alterations in the transcriptome profiles of infected groups based on their disease onset.

#### Longitudinal Analysis of Early and Later Moderate Stages of SARS-CoV-2 Infected Groups

**FIGURE 6:**
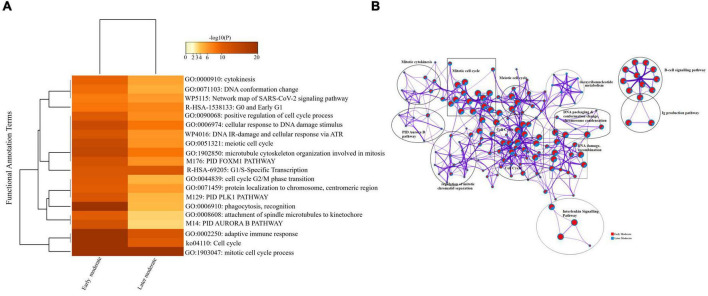
Longitudinal analysis of early and later moderate stages of SARS-CoV-2 infected groups. **(A)** Heat map displaying the top 20 enrichment clusters for early and later disease onset of moderate COVID-19 groups colored by -log10(P) values using a discrete color scale representing statistical significance. The functional annotation terms and their corresponding categories are M (canonical pathways), GO (Gene Ontology), ko (KEGG ontology), WP (Wiki pathways), R-HSA (Reactome Gene Sets), and hsa (KEGG pathway). **(B)** Network visualization of the enriched clusters for the list of DEGs generated by Metascape. The clusters were labeled manually. The pie chart color code depicts the gene lists’ identities, where the red color represents early moderate patients, and the blue color represents later moderate patients.

Firstly, we compared the DEGs among the group of moderate patients. The top 20 enriched annotation terms demonstrated that the moderate group of patients who were diagnosed at the early stage of disease onset (Group 1) was functionally annotated to the mitotic cell cycle process (GO: 1903047), cytokinesis (GO: 0000910), adaptive immune response (GO: 0002250) and phagocytosis (GO: 0006910) based on higher –log10(P) value. Therefore, early moderate patients exhibited aberrant host immune response to a greater extent than the moderate (Group 2) patients’ with the later disease onset (Figure 6A). Enrichment network visualization revealed that later moderate patients had the highest number of upregulated genes and their related GO terms compared to the early moderate patients. The regulatory pathways and clusters, including PID Aurora Pathway, cytokinesis, B-cell signaling pathway, Ig production pathway, and Interleukin signaling pathway, were evenly distributed among early and later disease onset of the moderate COVID-19 patients (Figure 6B). The Metascape generated heat map illustrating the functionally annotated enriched terms of the top 100 clusters for the moderate group is provided in Supplementary Figure 8A.

Interactome analysis of the protein-protein interaction (PPI) network derived from MCODE revealed several significant interactions among early and later moderate patients’ genes, including *TNF, CXCL2, IL-32, CCL3, TK1, CCNB2, CCL3L1, CDK1*, and *CDC20* as crucial genes of the early and later moderate disease onset (Supplementary Figure 6 and Supplementary File 5).

#### Longitudinal Analysis of Early and Later Critical Stages of SARS-CoV-2 Infected Groups

**FIGURE 7:**
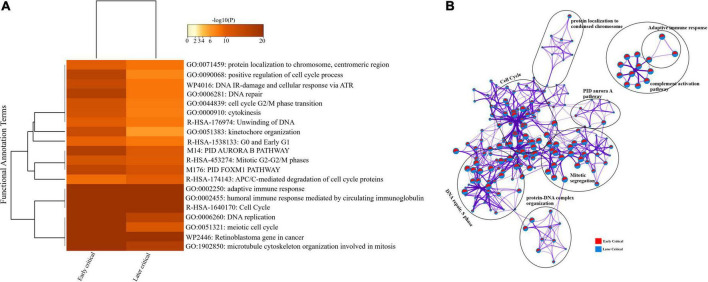
Longitudinal analysis of early and later critical stages of SARS-CoV-2 infected groups. **(A)** Heat map displaying the top 20 enrichment clusters for early and later disease onset of critical COVID-19 groups colored by -log 10 (P) values using a discrete color scale representing statistical significance. The functional annotation terms and their corresponding categories are M (canonical pathways), GO (Gene Ontology), WP (Wiki pathways), R-HSA (Reactome Gene Sets), and hsa (KEGG pathway). **(B)** Network visualization of the enriched clusters for the list of DEGs generated by Metascape. The clusters were labeled manually. The pie chart color code describes gene lists’ identities, where the red color represents early critical patients, and the blue color represents later critical patients.

Similarly, we compared the DEGs among the early and later disease onset of the critical COVID-19 patients. The functional enrichment analysis elucidated that the upregulated DEGs of both groups were evenly enriched in annotation terms, including; adaptive immune response (GO: 0002250), humoral immune response mediated by circulating immunoglobulin (GO: 0002455), and cytokinesis (GO: 0000910) (Figure 7A). Enrichment network visualization shows that processes such as adaptive immune response, complement activation pathway, and PID Aurora Pathway were evenly distributed among the early and later disease onset stages of critical COVID-19 groups (Figure 7B). The Metascape generated heat map illustrating the functionally annotated enriched terms of the top 100 clusters for the moderate group is provided in Supplementary Figure 8B.

The complex PPI networks revealed multiple complex interactions among genes, including *IFI27, CDC20, CDC6, CCR10, IGHG2, IGHV4-31*, and several other genes (Supplementary Figure 7 and Supplementary File 6).

### Cytokines Related Inflammatory Response Across COVID-19 Severity and the Onset

The significantly upregulated cytokines-related genes were screened from the cohort data and compared across each infected group based on their presence (Table 3). The cytokine-associated genes were produced unevenly during the SARS-CoV-2 progression based on the disease severity. C-X-C-chemokine ligands; *CXCL10*, *CXCL11*, and *CXCR3* were only upregulated in early critical patients and absent in other patients. In comparison, *CXCL2* was only upregulated in the early moderate stage. *IL32* The C-C-chemokine ligands; *CCL3L1, CCL3, CCL20, CCL4*, and *CCL4L2* were only upregulated in the early moderate stage. This finding suggests that these C-C-chemokine ligands can be further tested as biomarkers for early-stage disease patients. *IL7, IL17RC*, and *IFNLR1* were upregulated in the early stages of COVID-19 while absent in later disease stages. The *TNF* response was detected in all the groups except the later critical stage of patients. We suggested that high production of *TNF, TNFRSF13B*, and *IL32* might be associated with and can serve as markers for COVID-19 severity. Based on the disease onset and severity, it is suggested that in our patients’ data, *TNF* expression was highly elevated among critical patients at early diagnosis of the disease compared to the moderate patients at the late stage of disease diagnosis. *IL-32* was elevated in all patient groups, suggesting it to be a potential gene for producing cytokines-induced inflammatory storms regardless of the disease severity and onset. Several cytokine-induced inflammatory response-related genes were observed across each infected stage.

**TABLE 3:** Comparison of upregulated cytokine-storm-related genes in all groups of patients.

Number	Upregulated cytokines	Group1 Early moderate	Group2 Later moderate	Group3 Early critical	Group4 Later critical
1.	TNF	Yes	Yes	Yes	No
2.	TNFRSF13B	Yes	No	Yes	Yes
3.	TNFRSF17	No	No	Yes	No
4.	TNFRSF4	No	No	No	Yes
5.	TNFRSF21	Yes	No	No	No
6.	CXCL10	No	No	Yes	No
7.	CXCL11	No	No	Yes	No
8.	CXCR3	No	No	Yes	No
9.	CXCL2	Yes	No	No	No
10.	CCL3L1	Yes	No	No	No
11.	CCL3	Yes	No	No	No
12.	CCL20	Yes	No	No	No
13.	CCL4	Yes	No	No	No
14.	CCL4L2	Yes	No	No	No
15.	IL32	Yes	Yes	Yes	Yes
16.	IL2RB	Yes	Yes	No	No
17.	IL7	Yes	No	Yes	No
18.	IL17RC	Yes	No	Yes	No
19.	IFNLR1	Yes	No	Yes	No

## Discussion

In early 2020, the WHO (World Health Organization) declared the SARS-CoV-2 epidemic, and experts worldwide began looking for ways to manage patients and treat them effectively. The primary cause of mortality is the fast escalation of a severe pulmonary inflammatory response, subsequent tissue damage, fibrosis, and dysregulated host immunological response. Another critical cause of prevailing COVID-19 infections is the shutdown of host protein synthesis, a frequent viral infection hallmark. Besides cell-based therapeutics, various pharmaceutical alternatives have been developed to tackle SARS-CoV-2 infections. Thousands of researchers and laboratories have actively responded to this threat, generating vast amounts of biological data and biomedical information that computational biologists can use to define molecular disease bases, virus propagation, and development and identify potential treatments and vaccines.

On the cutting edge of medical science, nanotechnology has enabled tailored gene delivery systems, targeted drug delivery systems, imaging, biosensor platforms, and SARS-CoV-2 infection diagnostics (Gage et al., 2021). Recent research indicates that mRNA translational vaccines elicit more favorable humoral responses in the host than conventional drugs (Eren Sadioğlu et al., 2021). Recently, eight COVID-19 vaccines based on diverse technologies and formulations, including nanopharmaceuticals, have been authorized and approved for emergency use. The mRNA vaccines include; mRNA-1,273 from Moderna and BNT162 from Pfizer/BioNTech, the virus-inactivated Covaxin vaccine manufactured by Indian Barhat Biotech, the CoronaVac vaccine by Sinovac Biotech, China, the Ad26-based viral vector vaccine synthesized by Johnson and Johnson, the human adenovirus-based Sputnik V vaccine from the Gamaleya National Center of Epidemiology and Microbiology, Russia and the chimpanzee adenovirus-based AZD1222 (Covidshield) vaccine from Oxford-Astra Zeneca (Bidram et al., 2021; Mishra and Tripathi, 2021). The development of novel SARS-CoV-2 variants with high morbidity, mortality, and transmissibility has posed a new challenge for these vaccines, highlighting the need for new vaccine formulations with high efficacy (Gage et al., 2021). Nanotechnologists and omics-data analysts should work together to understand molecular biology, data analysis, and data visualization to utilize innovative technologies to combat deadly pathogens.

Recent advances in multi-omics methods, including proteomics, genomics, metabolomics, total RNA sequencing, transcriptomics, and single-cell transcriptomics, have made comprehending the pathogen and disease (Wang et al., 2021; Zhang et al., 2021). Therefore, we employed an RNA-sequencing-based approach to identify new therapeutic vaccines and drug targets to diagnose and inhibit SARS-CoV-2 infections. The preliminary studies of SARS-CoV-2 patients having severe respiratory disorders and failure in respiratory tracts divulged a dysregulated host immune response, hyper-inflammatory responses, and lymphopenia (Unterman et al., 2022). It is suggested that blood acts as the potential remote biosensor to reflect the multifaceted variations arising in the immune system and other cells and highly infected tissues in PBMCs (Sadanandam et al., 2020) to explain system-wide changes, disease progression, and onset in COVID-19 patients (Huang et al., 2020; Liao et al., 2020; Sadanandam et al., 2020). Our study sequenced and analyzed the four PBMC transcriptomes of SARS-CoV-2 infected groups to identify the key regulators and DEGs responsible for the dysregulated host adaptive immune response. Each group was specified based on their disease severity and the onset (Methods). Our experimental design allowed us; (1) to sequence profile the pooled PBMCs transcriptome of all infected samples used in the current study, (2) to quantify the expression profile of SARS-CoV-2 infected groups, (3) to evaluate the DEGs in comparison with the healthy controls, (4) to determine the global transcriptome profile in cohort data (5) to compare the DEGs across disease severity and the onset (6) new potential therapeutic and vaccine targets to combat SARS-CoV-2 infections. The current systems biology method applying differential gene expression analysis pipeline manifested the progressive dynamics of the host immune responses toward this deadly disease based on severity and onset. It has underlined the unique and diverse hallmarks of the host immune system that distinguish moderate and critical SARS-CoV-2 patients. The immunological regulatory networks identified in this study have the potential to improve and improve the understanding of the numerous host-pathogen interactions that promote pathology and virulence in the early stages of disease.

The COVID-19 cohort’s differential gene expression analysis revealed global transcriptome variations across COVID-19 severity levels. It was observed that the adaptive immune response was dysregulated in the COVID-19 patients in comparison with the healthy control datasets. Measuring the antibody levels specific to SARS-CoV-2 in the blood, such as immunoglobin (Ig), provides not only an alternative method for diagnosis and treatment of SARS-CoV-2 infections (including infected individuals) but also a simple way to measure adaptive immunity in convalescent patients or after vaccination (Ma et al., 2020). The high level of antibodies specific to SARS-CoV-2, specifically those which can bind and neutralize the virus, would strongly indicate that an immunized host could resist SARS-CoV-2 infection. We identified several upregulated immunoglobin genes *(IGLV9-49, IGHV7-4, IGHV3-64, IGHV1-24, IGKV1D-12*, and *IGKV2-29)* for dysregulation of the antibody-mediated host immune responses. As a result, antibody-based therapies will continue to be important in treating and eventually preventing COVID-19.

Our results identified the elevated expression of MSCs (Mesenchymal stem cells genes, including; *KIF14, KIF4A, UCHL1, KIFCI, MYBL2*, and *CDC20*, contributing to dysregulated cell cycle phases and DNA proliferations. Such cells are necessary for tissue repair and can also influence the pulmonary environment by paracrine production of various mediators. They can control or enhance inflammation, drive the development of other stem cells, and limit the viral burden (Henriques-Pons et al., 2022). Consistent with the recent finding (Henriques-Pons et al., 2022), our results demonstrate that protein-encoding genes associated with these cells could be a promising therapeutic alternative against SARS-CoV-2 infections.

We have also identified the upregulated ribosomal proteins *(RPL29, RPL4P2, RPL5*, and *RPL14).* Some studies have indicated that various host factors must be used to compete with the infectious process, including Ribosomal proteins (RPs). They can either inhibit viral replication by binding to certain phosphoproteins or activate host immune responses (Rofeal and El-Malek, 2020). Studies on SARS-CoV have implicated *NSP1* (non-structural protein 1) as a critical factor in host translation shutdown. In infected cells or upon its ectopic expression, NSP1 inhibits human translation by binding with human ribosomal subunits disrupting the translational mechanism. In the current study, 60S Ribosomal protein *RPL29* protein was highly expressed across all disease stages. It is a component of functionally stable ribosomes and plays a vital role in protein synthesis (DeLabre et al., 2002; Kirn-Safran et al., 2007). *RPL29-*deficient embryonic fibroblasts proliferate and synthesize proteins at a slower pace (Jones et al., 2013). According to our findings, future studies should look at the efficacy of anti-RPL29 mRNA-based nanomedicines and therapies in combination with chemotherapeutics against SARS-CoV-2 infections.

Inflammation is a double-edged sword in viral pneumonia. Even if beneficial inflammation is required to fight infections in adjacent tissues, exaggerated inflammatory reactions result in excessive inflammatory cytokine production, which has negative consequences such as progressive respiratory failure and various organ failures (Xiong et al., 2020). The critical factor in SARS-CoV-2 infections could be reducing antiviral defense associated with the innate immune response and the higher expression of inflammatory cytokines (Costela-Ruiz et al., 2020). In the limited timeframe after the COVID-19 emergence, various studies have identified increased expression of several cytokines and chemokines in most patients (Chen C. et al., 2020; Huang et al., 2020; Liu K. et al., 2020; Liu Y. et al., 2020; Qin et al., 2020; Wang W. et al., 2020; Xu et al., 2020). Although our findings corroborate previous studies, some genes implicated in cytokine inflammatory response identified in our study were unique. Our results demonstrated the elevated expression of inflammatory cytokines and chemokines, including; Tumor Necrosis Factor *(TNF, TNFRSF17*, and *TNFRSF13B*), Interleukin signaling pathways, C-C motif chemokine ligands *(CCL3, CCL25, CCL4L2, CCL22*, and *CCL4)*, C-X-C motif chemokine ligands *(CXCL2, CXCL10*, and *CXCL11)*. These genes are also responsible for the activation of receptor-associated cytokine inflammatory responses. This finding corroborates other studies representing the mononuclear blood cell population in lung tissues of COVID-19 patients (Vabret et al., 2020). We suggest that high production of *TNF, TNFRSF13B*, and *IL32* might be associated with and serve as markers for COVID-19 severity-specific therapies. *IL-32* was elevated in all infected groups, suggesting that it can be the potential gene for producing the cytokines-induced inflammatory storm at each stage of the COVID-19 disease progression. *IL-32* can affect several physiological and cellular functions, including survival inflammation, cell death, and response toward certain pathogens like Leishmania, HIV, and Mycobacterium. A recent study has proposed the role of *IL-32* in chronic inflammatory diseases such as airway and lung diseases, including COPD (Chronic obstructive pulmonary disease) (Gautam and Pandit, 2021). Therefore, this unique intracellular cytokine can be explored at protein levels to verify its role during SARS-CoV-2 infection.

In conclusion, our study highlights key functional and molecular pathways implicated in SARS-CoV-2 pathogenesis and identifies a distinct expression pattern linked to SARS-CoV-2-related critical illness outcomes. This research will contribute to understanding the host immune response during SARS-CoV-2 progression and can help illuminate the COVID-19 infectious pathways and provide a foundation for designing rational immunotherapies. However, the functional importance of the therapeutic targets identified in this study remains demonstrated by *in vivo, in vitro, and in silico* approaches. However, nanobiotechnologists and pharmaceutical scientists can consider the proposed medicinal drugs and vaccine targets to design more effective and efficient drug delivery systems to combat ongoing SARS-CoV-2 infections in the future.

### Limitations, Challenges, and Recommendations for Future Research

The primary goal of this work was to use differential gene expression analysis to predict and find potential therapeutic possibilities for SARS-CoV-2 infection treatment. The current study’s limitations and constraints reflect various recommendations for future research to help understand this study more effectively. Firstly, our study mainly emphasizes the transcriptome analysis of PBMCs in blood by using the RNA sequencing method. The alterations in gene expression at the protein level should also be verified. Additionally, the qPCR technique can be carried out to confirm the results found from the sequencing, where a smaller number of genes can be identified for a higher number of samples.

The challenges encountered in this work show that there is currently no validation of RNA sequencing data to identify changes in PBMC phenotype at the protein level. In the future, the assessment of protein expression within cells isolated from infected patients can be done using flow cytometry or similar techniques to denote cell types comprising PBMCs to observe gross genotype changes. The suggested novel targets in this study can be verified and further investigated by *in vivo* and *in vitro* studies at the protein levels.

Immune cell data from lesion locations such as bronchoalveolar lavage fluid and lungs and data from various drug delivery systems could make this study more extensive and conclusive. In summary, this study’s data-driven research of transcriptomics data can be compared with already published multi-omics data, which may help determine the associations between immune response and disease outcome.

## Data Availability Statement

The test (infected) raw sequencing datasets have been submitted to BIG Data GSA and can be found under the following link: https://ngdc.cncb.ac.cn/gsa-human/browse/HRA002526. The control datasets presented in this study can be found in the online repositories as mentioned in the Methods section.

## Ethics Statement

The studies involving human participants were reviewed and approved by the Medical Ethical Committee of The First Affiliated Hospital of the University of Science and Technology of China (USTC) (approval number 2020-XG (H)-199 019). The patients/participants provided their written informed consent to participate in this study.

## Author Contributions

ZK conceived the presented idea, extracted and analyzed the data, wrote the original draft, and formatted the manuscript for submission. MH and HH assisted in the experiments and sample collection. MSR assisted in writing and proofreading the manuscript. MA, ZN, WZ, and AK extensively reviewed, edited, and formatted the manuscript for submission. TJ conceptualized the main idea, provided resources in data extraction and financial assistance during the whole study, and supervised the entire research. All authors read and approved the final version of the manuscript for publication.

## Conflict of Interest

The authors declare that the research was conducted in the absence of any commercial or financial relationships that could be construed as a potential conflict of interest.

## Publisher’s Note

All claims expressed in this article are solely those of the authors and do not necessarily represent those of their affiliated organizations, or those of the publisher, the editors and the reviewers. Any product that may be evaluated in this article, or claim that may be made by its manufacturer, is not guaranteed or endorsed by the publisher.

## Footnotes

1
https://covid19.who.int/

2
https://www.omegabiotek.com/

3
https://www.ncbi.nlm.nih.gov/bioproject/PRJNA252189

4
https://ngdc.cncb.ac.cn/gsa/browse/CRA002390

5
https://github.com/agordon/fastx_toolkit

6
https://github.com/OpenGene/fastp

7
https://github.com/jstjohn/SeqPrep

8
https://github.com/najoshi/sickle

9
https://daehwankimlab.github.io/hisat2/

10
http://asia.ensembl.org/Homo_sapiens/Info/Index

11
https://ccb.jhu.edu/software/stringtie/

12
https://ccb.jhu.edu/software/stringtie/gffcompare.shtml

13
https://github.com/deweylab/RSEM

14
https://www.bioconductor.org/packages/release/bioc/html/NOISeq.html

15
https://www.bioconductor.org/packages/release/bioc/html/limma.html

16
http://bioconductor.org/packages/release/bioc/html/DESeq2.html

17
https://metascape.org/gp/index.html#/main/step1
