# Corrigendum: Identification of novel therapeutic candidates against SARS-CoV-2 infections: An application of RNA sequencing toward mRNA based nanotherapeutics

**DOI:** 10.3389/fmicb.2022.1059437

**Published:** 2022-10-21

**Authors:** Zunera Khalid, Ma Huan, Muhammad Sohail Raza, Misbah Abbas, Zara Naz, Arnaud John Kombe Kombe, Weihong Zeng, Hongliang He, Tengchuan Jin

**Affiliations:** ^1^Department of Obstetrics and Gynecology, The First Affiliated Hospital of University of Science and Technology of China (USTC), Division of Life Sciences and Medicine, University of Science and Technology of China, Hefei, China; ^2^CAS Key Laboratory of Genomic and Precision Medicine, Beijing Institute of Genomics, Chinese Academy of Sciences, Beijing, China; ^3^China National Center for Bioinformation, Beijing, China; ^4^University of Chinese Academy of Sciences, Beijing, China; ^5^CAS Key Laboratory of Innate Immunity and Chronic Disease, School of Basic Medical Sciences, Division of Life Sciences and Medicine, University of Science and Technology of China, Hefei, China; ^6^Department of Infectious Diseases, The First Affiliated Hospital of University of Science and Technology of China (USTC), Division of Life Sciences and Medicine, University of Science and Technology of China, Hefei, China; ^7^CAS Center for Excellence in Molecular Cell Science, Shanghai, China

**Keywords:** SARS-CoV-2, PBMCs, RNA sequencing, upregulated genes, mRNA based nanotherapeutics, COVID-19 management

In the published article, there was an error in affiliations 1, 5 and 6. Instead of “Division of Life Sciences and Medicine, Department of Obstetrics and Gynecology, The First Affiliated Hospital of University of Science and Technology of China (USTC), University of Science and Technology of China, Hefei, China,” it should be “Department of Obstetrics and Gynecology, The First Affiliated Hospital of University of Science and Technology of China (USTC), Division of Life Sciences and Medicine, University of Science and Technology of China, Hefei, China.”

Instead of “CAS Key Laboratory of Innate Immunity and Chronic Disease, Division of Life Sciences and Medicine, School of Basic Medical Sciences, University of Science and Technology of China, Hefei, China,” it should be “CAS Key Laboratory of Innate Immunity and Chronic Disease, School of Basic Medical Sciences, Division of Life Sciences and Medicine, University of Science and Technology of China, Hefei, China.”

Instead of “Division of Life Sciences and Medicine, Department of Infectious Diseases, The First Affiliated Hospital of USTC, University of Science and Technology of China, Hefei, China,” it should be “Department of Infectious Diseases, The First Affiliated Hospital of University of Science and Technology of China (USTC), Division of Life Sciences and Medicine, University of Science and Technology of China, Hefei, China.”

The authors apologize for this error and state that this does not change the scientific conclusions of the article in any way. The original article has been updated.

## Publisher's note

All claims expressed in this article are solely those of the authors and do not necessarily represent those of their affiliated organizations, or those of the publisher, the editors and the reviewers. Any product that may be evaluated in this article, or claim that may be made by its manufacturer, is not guaranteed or endorsed by the publisher.

