# Supplementary material for: Identification of Novel Therapeutic Candidates Against SARS-CoV-2 Infections: An Application of RNA Sequencing Toward mRNA Based Nanotherapeutics
Source: Front Microbiol. 2022 Aug 2;13:901848. doi: 10.3389/fmicb.2022.901848 (PMC9378778; doi:10.3389/fmicb.2022.901848)
Supplement: Supplementary file 1 [file Data_Sheet_1.zip › Supplementary_Material/Supplementary_Figure_S2.docx]

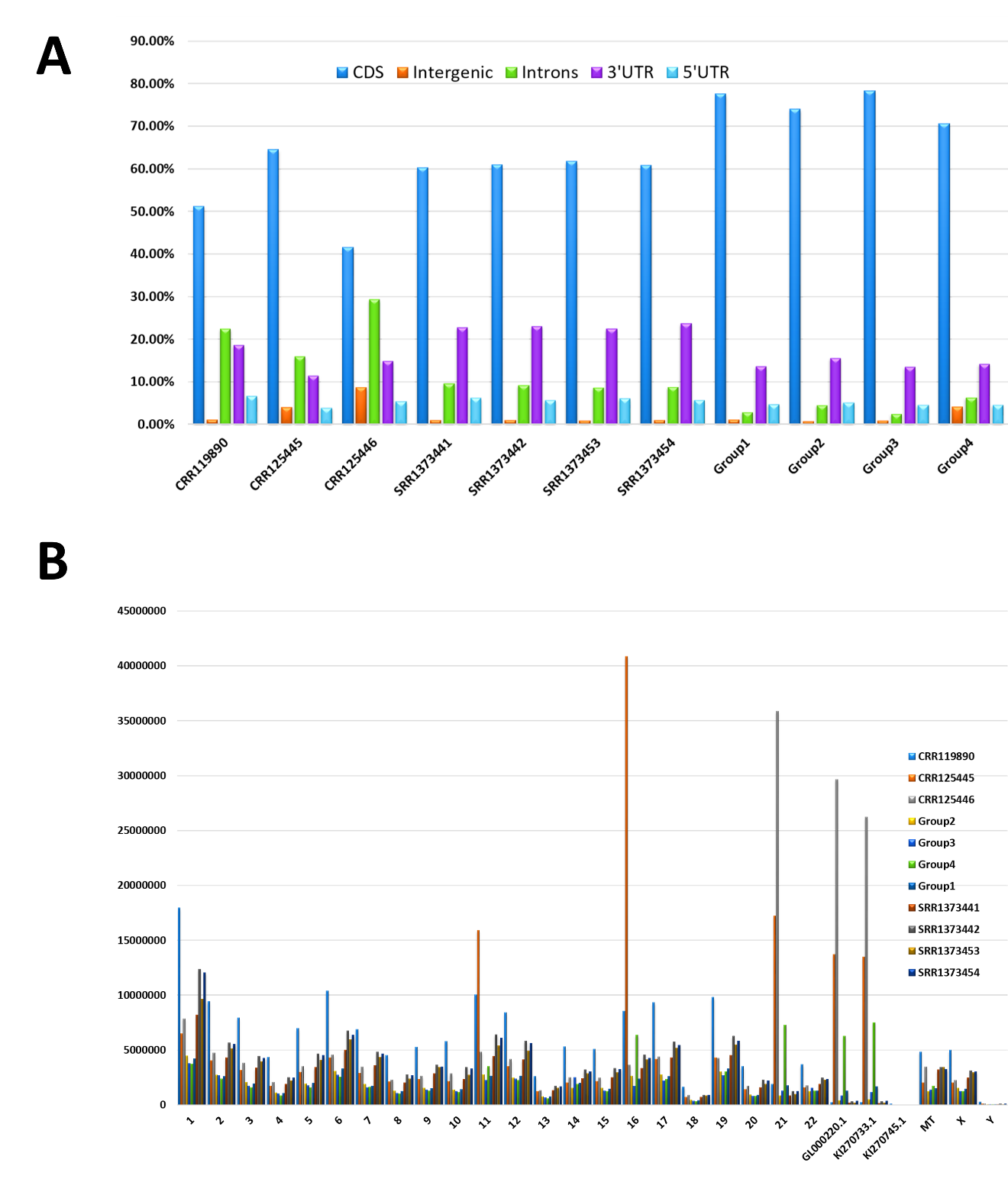


**Supplementary Figure S2.** Distribution of reads for each sample at different chromosomes of human genome
