# Supplementary material for: Identification of Novel Therapeutic Candidates Against SARS-CoV-2 Infections: An Application of RNA Sequencing Toward mRNA Based Nanotherapeutics
Source: Front Microbiol. 2022 Aug 2;13:901848. doi: 10.3389/fmicb.2022.901848 (PMC9378778; doi:10.3389/fmicb.2022.901848)
Supplement: Supplementary file 1 [file Data_Sheet_1.zip › Supplementary_Material/Supplementary_Figure_S4.docx]

**
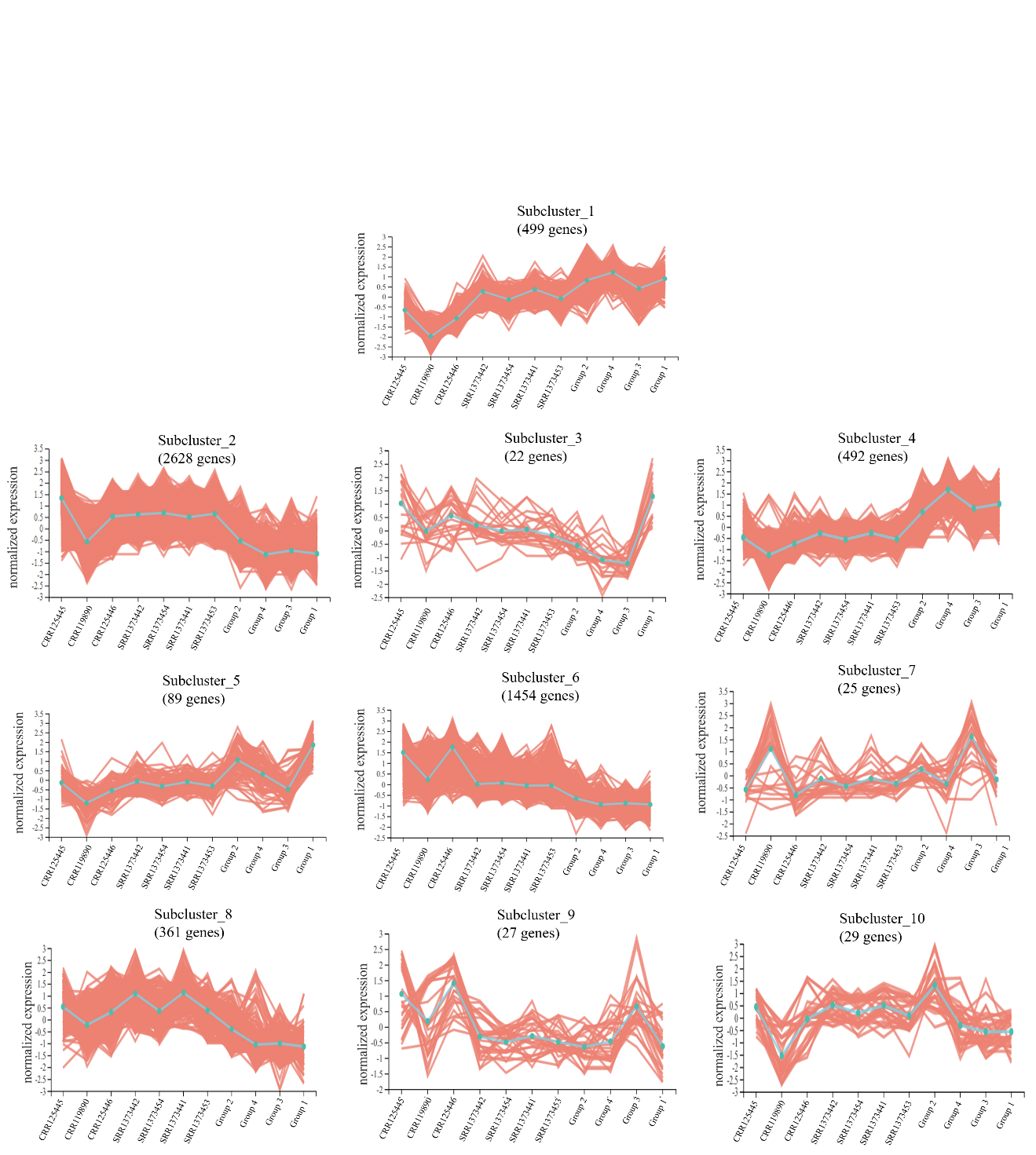
**

**Supplementary Figure S4.** Normalized expression patterns of genes in the ten main sub clusters, corresponding to the hierarchical clustering heat map as represented in **Figure 4A** of the main manuscript.
