# Supplementary material for: Identification of Novel Therapeutic Candidates Against SARS-CoV-2 Infections: An Application of RNA Sequencing Toward mRNA Based Nanotherapeutics
Source: Front Microbiol. 2022 Aug 2;13:901848. doi: 10.3389/fmicb.2022.901848 (PMC9378778; doi:10.3389/fmicb.2022.901848)
Supplement: Supplementary file 1 [file Data_Sheet_1.zip › Supplementary_Material/Supplementary_Figure_S5.docx]

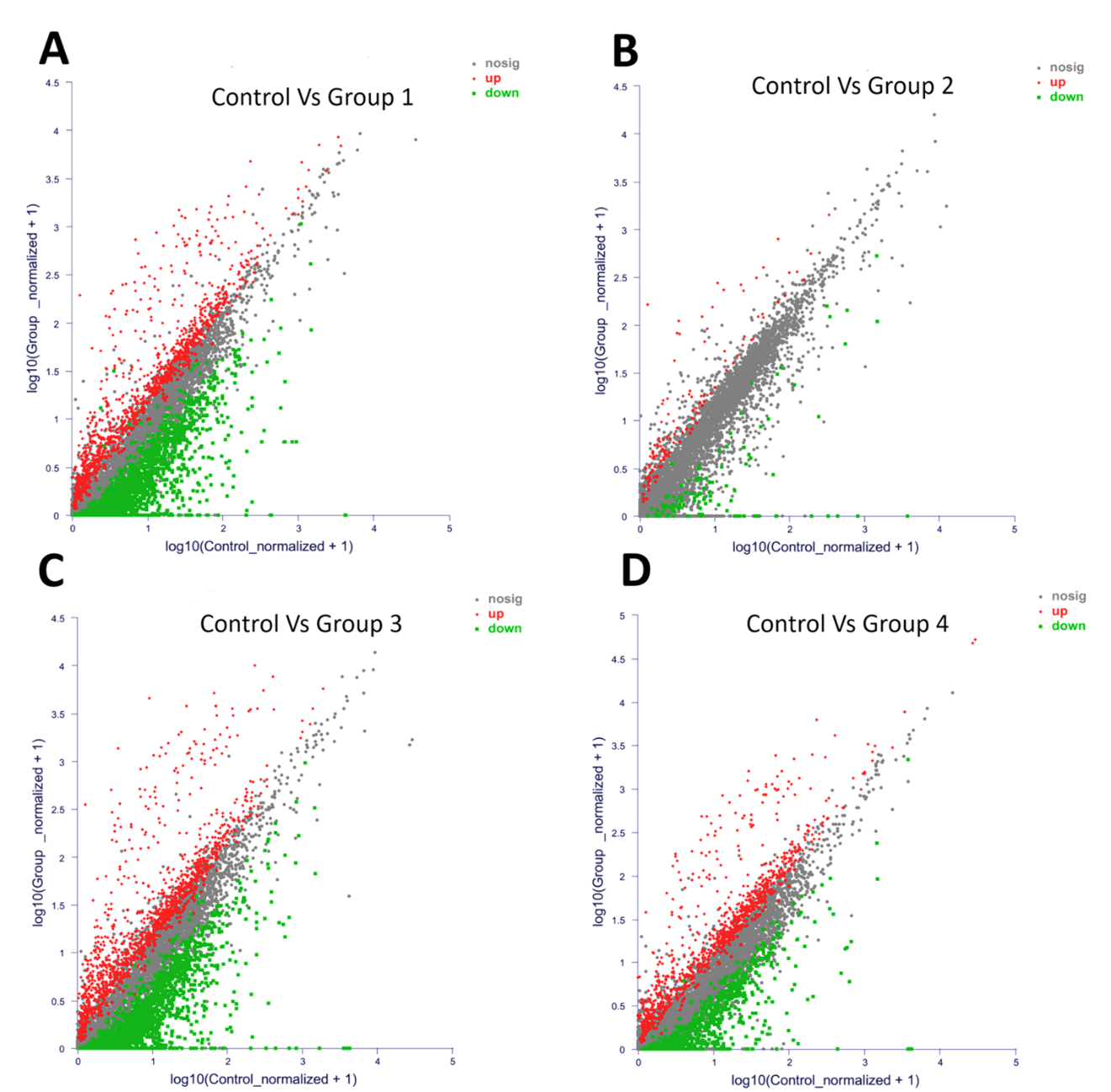


**Supplementary Figure S5.** Scatter plots showing the differentially expressed genes when comparing the four groups with the control cohort. The horizontal and vertical axes represent the log2(TPM) value of two group samples. Each point represents a gene, and the closer each point is to the origin, the lower the expression level. Red color represents upregulated genes, green color represents downregulated genes, and black color represents genes with no difference in expression.
