# Supplementary material for: Identification of Novel Therapeutic Candidates Against SARS-CoV-2 Infections: An Application of RNA Sequencing Toward mRNA Based Nanotherapeutics
Source: Front Microbiol. 2022 Aug 2;13:901848. doi: 10.3389/fmicb.2022.901848 (PMC9378778; doi:10.3389/fmicb.2022.901848)
Supplement: Supplementary file 1 [file Data_Sheet_1.zip › Supplementary_Material/Supplementary_Figure_S7.docx]

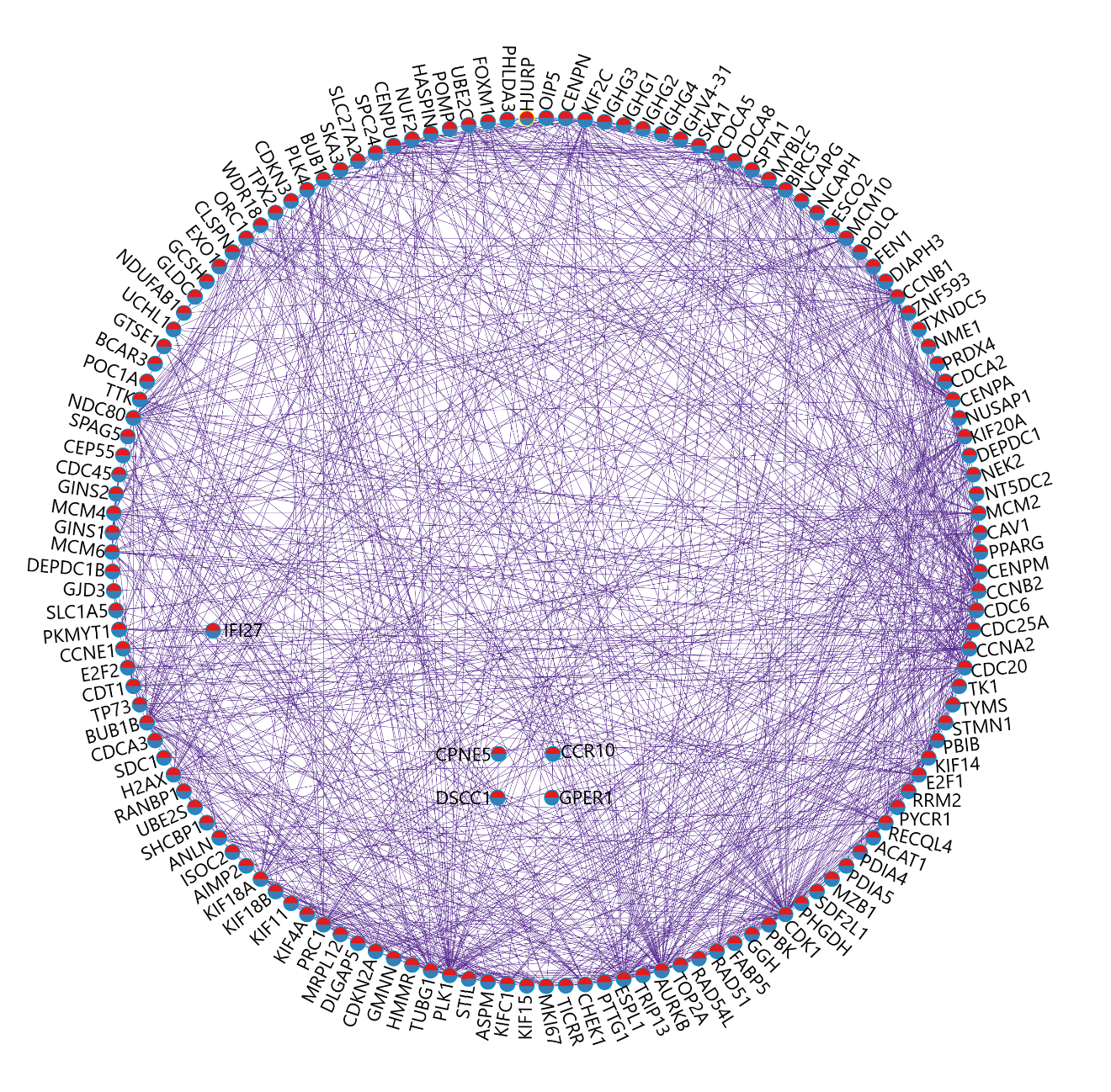


**Supplementary Figure S7.** Selected MCODE components were recognized to draw an interactome diagram from the combined list of top DEGs from early critical and later critical group of patients. Each node represents a protein with a pie chart encoding its presence in each group. The red color represents early critical group and blue color represents later critical groups.
