# Supplementary material for: Identification of Novel Therapeutic Candidates Against SARS-CoV-2 Infections: An Application of RNA Sequencing Toward mRNA Based Nanotherapeutics
Source: Front Microbiol. 2022 Aug 2;13:901848. doi: 10.3389/fmicb.2022.901848 (PMC9378778; doi:10.3389/fmicb.2022.901848)
Supplement: Supplementary file 1 [file Data_Sheet_1.zip › Supplementary_Material/Supplementary_Figure_S8.docx]

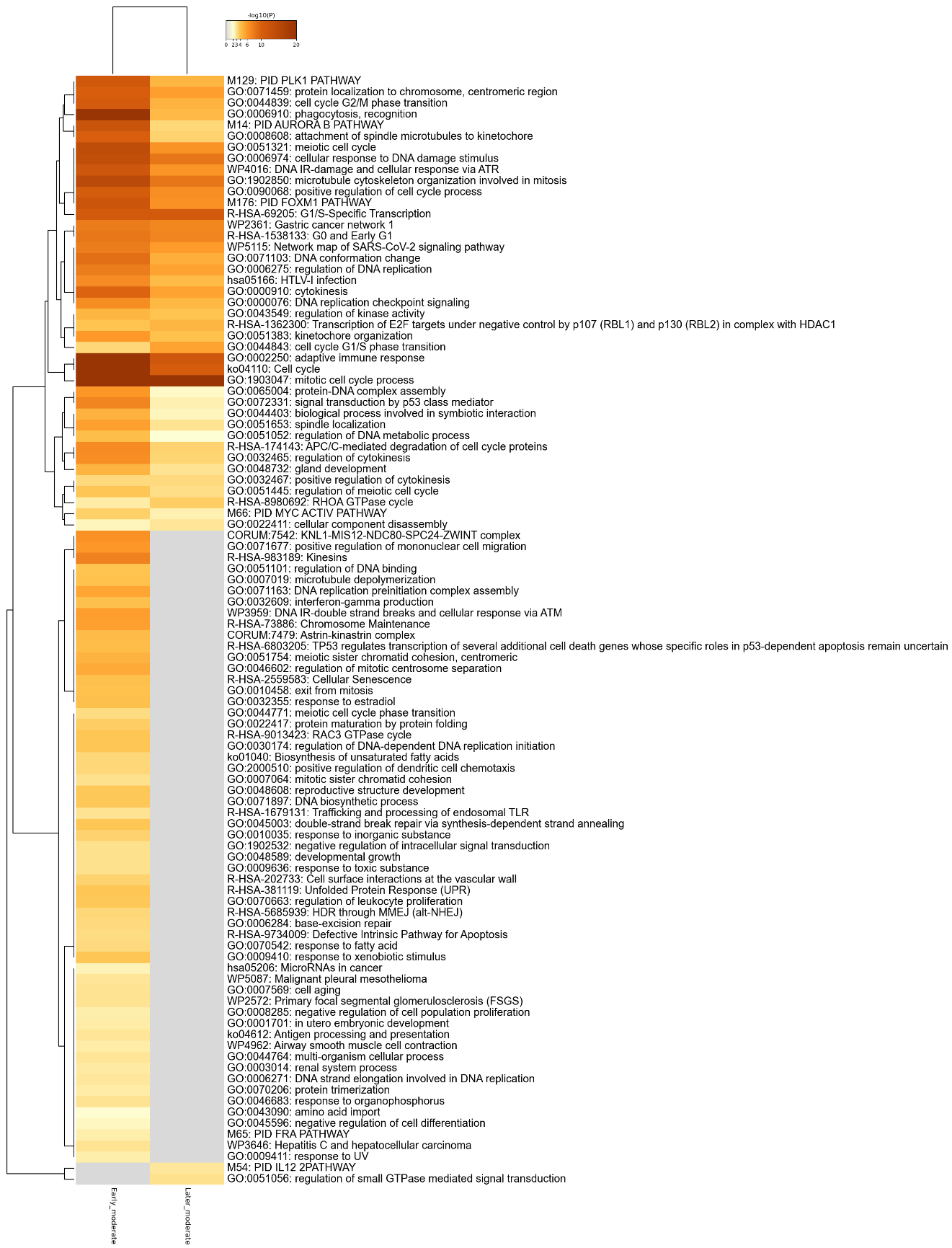


**Supplementary Figure S8_A.** Heat map displaying the top 100 enriched clusters for early and later moderate patients using a distinct color scale to represent statistical significance using -log 10(P). The functional annotation terms and their categories are M (canonical pathways), GO (Gene Ontology), WP (Wiki pathways), R-HSA (Reactome Gene Sets), and hsa (KEGG pathway). Gray color designates the lack of significance.


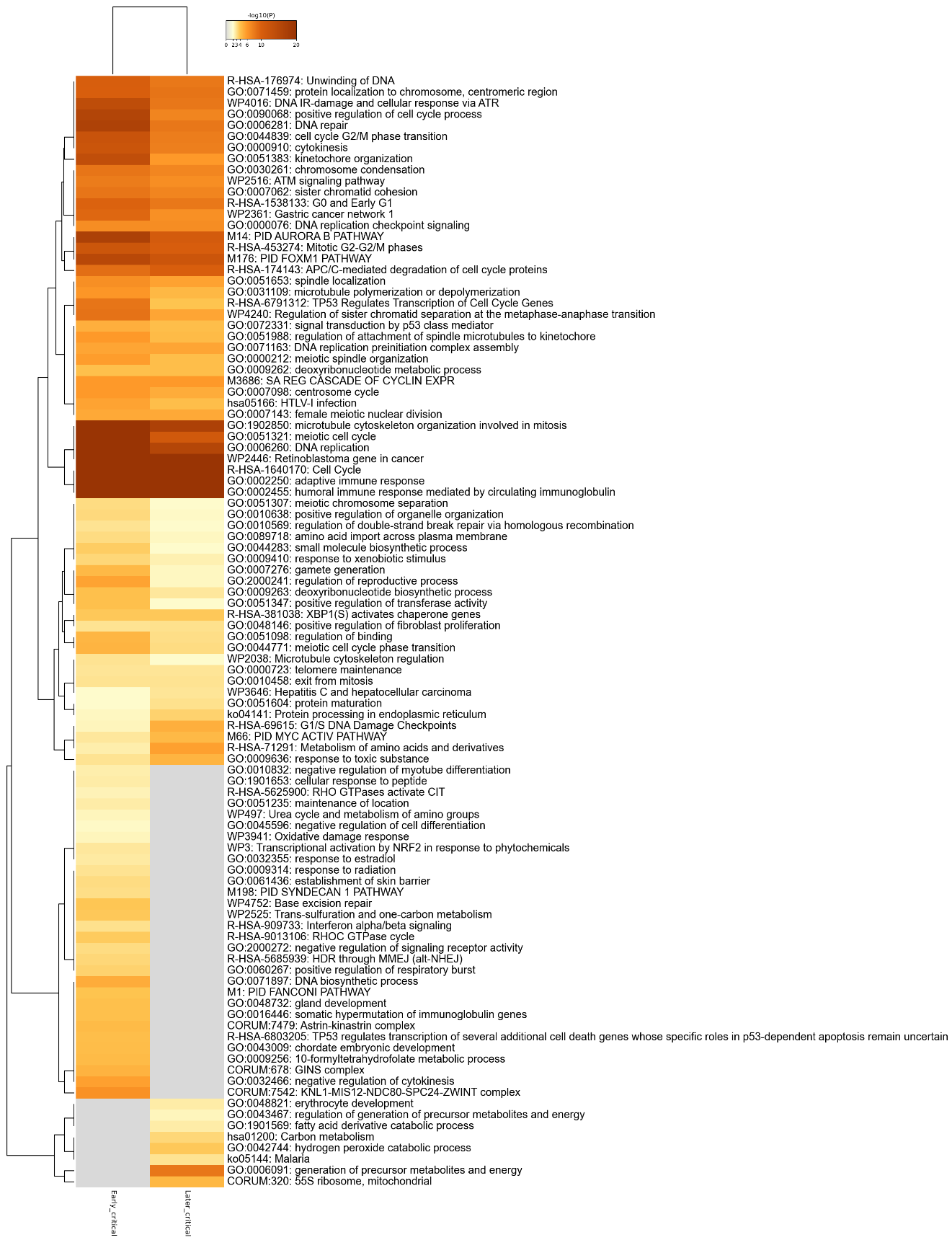


**Supplementary Figure S8_B.**  Heat map displaying the top 100 enriched clusters for early and later critical patients using a distinct color scale to represent statistical significance using -log 10(P). The functional annotation terms and their categories are M (canonical pathways), GO (Gene Ontology), WP (Wiki pathways), R-HSA (Reactome Gene Sets), and hsa (KEGG pathway). Gray color designates the lack of significance.
