# Supplementary material for: Identification of Novel Therapeutic Candidates Against SARS-CoV-2 Infections: An Application of RNA Sequencing Toward mRNA Based Nanotherapeutics
Source: Front Microbiol. 2022 Aug 2;13:901848. doi: 10.3389/fmicb.2022.901848 (PMC9378778; doi:10.3389/fmicb.2022.901848)
Supplement: Supplementary file 1 [file Data_Sheet_1.zip › Supplementary_Material/Supplementary_File_S1.docx]

**Supplementary File S1**

**Patients Information and RNA sample pooling information for each group**

**Patients Classification:**

According to the "New Coronavirus Pneumonia Diagnosis and Treatment Plan (Trial Sixth Edition)": The clinical classification of new coronavirus pneumonia includes:

1. **Moderate**: symptoms such as fever and respiratory tract inflammation, and pneumonia can be seen on imaging.
2. **Critical/severe**: Meet any of the following: 1. Shortness of breath, breathing rate ≥ 30 times/min; 2. In resting state, oxygen saturation ≤ 93%; 3. Arterial blood oxygen partial pressure PaO2/oxygen concentration FiO2≤300mmHg. 4. Lung imaging showed that the lesions progressed significantly within 24-48 hours> 50% were treated as severe. 5. Had Respiratory failure and mechanical ventilation.

The information of twenty patients divided into four RNA pools based on disease and severity is given below in tabular form for each group.

**Table 1:** Patients information for RNA pool 1 **(Group 1)** having **early moderate** patients based on disease onset

| **Patient No.** | **Age** | **Sex** | **Disease severity** | **Collection date** | **Days after illness onset** |
| --- | --- | --- | --- | --- | --- |
| 1 | 50 | female | Moderate | 200220 | 10 |
| 2 | 31 | female | Moderate | 200220 | 15 |
| 3 | 66 | female | Moderate | 200220 | 10 |
| 4 | 35 | female | Moderate | 200220 | 20 |
| 5 | 47 | female | Moderate | 200220 | 20 |
| **Total patients=5** | **Average age=45** |  |  |  | **Average disease onset= 15** |

**Table 2:** Patients information for RNA pool 2 **(Group 2)** having **later moderate** patients based on disease onset

| **Patient No.** | **Age** | **Sex** | **Disease severity** | **Collection date** | **Days after illness onset** |
| --- | --- | --- | --- | --- | --- |
| 6 | 35 | female | Moderate | 200226 | 27 |
| 7 | 50 | female | Moderate | 200226 | 27 |
| 8 | 49 | male | Moderate | 200226 | 30 |
| 9 | 53 | female | Moderate | 200226 | 31 |
| 10 | 45 | female | Moderate | 200226 | 28 |
| **Total patients=5** | **Average age=46** |  |  |  | **Average disease onset= 29** |

**Table 3:** Patients information for RNA pool 3 **(Group 3)** having early critical patients based on disease onset

| **Patient No.** | **Age** | **Sex** | **Disease severity** | **Collection date** | **Days after illness onset** |
| --- | --- | --- | --- | --- | --- |
| 11 | 88 | male | Critical | 200217 | 14 |
| 12 | 39 | male | critical | 200217 | 13 |
| 13 | 78 | male | Severe | 200217 | 18 |
| **Total patients=3** | **Average age=68** |  |  |  | **Average disease onset= 15** |

**Table 4:** Patients information for RNA pool 4 (Group 4) having early moderate patients based on disease onset

| **Patient No.** | **Age** | **Sex** | **Disease severity** | **Collection date** | **Days after illness onset** |
| --- | --- | --- | --- | --- | --- |
| 14 | 40 | male | severe | 200219 | 32 |
| 15 | 63 | male | Critical | 200219 | 20 |
| 16 | 56 | female | Critical | 200219 | 24 |
| 17 | 47 | male | severe | 200219 | 31 |
| 18 | 65 | female | Critical | 200219 | 21 |
| 19 | 68 | male | Critical | 200219 | 30 |
| 20 | 91 | male | Critical | 200219 | 20 |
| **Total patients=5** | **Average age=62** |  |  |  | **Average disease onset= 25** |
