# Supplementary material for: Identification of Novel Therapeutic Candidates Against SARS-CoV-2 Infections: An Application of RNA Sequencing Toward mRNA Based Nanotherapeutics
Source: Front Microbiol. 2022 Aug 2;13:901848. doi: 10.3389/fmicb.2022.901848 (PMC9378778; doi:10.3389/fmicb.2022.901848)
Supplement: Supplementary file 1 [file Data_Sheet_1.zip › Supplementary_Material/Supplementary_Table_1.docx]

**Table 1:** Quality Control statistics for sequencing data

| **Condition** | **Sample** | **Raw reads** | **Raw bases** | **Clean reads** | **Clean bases** | **Error rate (%)** | **Q20 (%)** | **Q30 (%)** | **GC content (%)** |
| --- | --- | --- | --- | --- | --- | --- | --- | --- | --- |
|  | **CRR119890** | 1.56E+08 | 1.56E+10 | 1.53E+08 | 1.52E+10 | 0.03 | 97.93 | 91.6 | 49.13 |
|  | **CRR125445** | 97158490 | 1.46E+10 | 96010278 | 1.39E+10 | 0.0243 | 98.27 | 94.96 | 52.6 |
|  | **CRR125446** | 97583782 | 1.46E+10 | 95686322 | 1.38E+10 | 0.0246 | 98.1 | 94.58 | 50.89 |
| Control | **SRR1373441** | 70743726 | 3.47E+09 | 69178590 | 3.38E+09 | 0.0137 | 99.59 | 98.21 | 49.73 |
|  | **SRR1373442** | 96487488 | 4.73E+09 | 94549298 | 4.62E+09 | 0.0155 | 99.28 | 97.06 | 49.85 |
|  | **SRR1373453** | 83745270 | 4.1E+09 | 81973986 | 4E+09 | 0.015 | 99.35 | 97.32 | 50.17 |
|  | **SRR1373454** | 92833080 | 4.55E+09 | 90738430 | 4.43E+09 | 0.0157 | 99.26 | 96.96 | 49.53 |
|  | **Group 1** | 45521292 | 6.87E+09 | 44342340 | 6.63E+09 | 0.0251 | 97.95 | 94.04 | 51.75 |
|  | **Group 2** | 45821982 | 6.92E+09 | 44683536 | 6.62E+09 | 0.0244 | 98.25 | 94.79 | 50.56 |
| Infected | **Group 3** | 41330562 | 6.24E+09 | 40351760 | 6.04E+09 | 0.0254 | 97.84 | 93.78 | 51.48 |
|  | **Group 4** | 46510268 | 7.02E+09 | 45559188 | 6.81E+09 | 0.0253 | 97.9 | 93.92 | 52.75 |
