# Supplementary material for: Identification of Novel Therapeutic Candidates Against SARS-CoV-2 Infections: An Application of RNA Sequencing Toward mRNA Based Nanotherapeutics
Source: Front Microbiol. 2022 Aug 2;13:901848. doi: 10.3389/fmicb.2022.901848 (PMC9378778; doi:10.3389/fmicb.2022.901848)
Supplement: Supplementary file 1 [file Data_Sheet_1.zip › Supplementary_Material/Supplementary_Table_2.docx]

**Supplementary Table 2**

**Supplementary Table 2**: Statistics of Mapped reads with the human genome.

| **Sample** | **Sample** | **Total reads** | **Total mapped** | **Multiple mapped** | **Uniquely mapped** |
| --- | --- | --- | --- | --- | --- |
| Control | CRR119890 | 152935346 | 149449283(97.72%) | 4898394(3.2%) | 144550889(94.52%) |
|  | CRR125445 | 96010278 | 93618169(97.51%) | 22150337(23.07%) | 71467832(74.44%) |
|  | CRR125446 | 95686322 | 92769671(96.95%) | 20715523(21.65%) | 72054148(75.3%) |
|  | SRR1373441 | 69178590 | 66492337(96.12%) | 3930255(5.68%) | 62562082(90.44%) |
|  | SRR1373442 | 94549298 | 90997568(96.24%) | 5077902(5.37%) | 85919666(90.87%) |
|  | SRR1373453 | 81973986 | 78919329(96.27%) | 4651232(5.67%) | 74268097(90.6%) |
|  | SRR1373454 | 90738430 | 87723592(96.68%) | 4780037(5.27%) | 82943555(91.41%) |
| Infected | Group 1 | 44342340 | 42369847(95.55%) | 2409221(5.43%) | 39960626(90.12%) |
|  | Group 2 | 44683536 | 42057070(94.12%) | 1715802(3.84%) | 40341268(90.28%) |
|  | Group 3 | 40351760 | 38070675(94.35%) | 1998665(4.95%) | 36072010(89.39%) |
|  | Group 4 | 45559188 | 43383595(95.22%) | 6872884(15.09%) | 36510711(80.14%) |
